# Geographical Heterogeneity in Antimalarial Resistance Markers by Genomic Surveillance in Angola, 2023

**DOI:** 10.4269/ajtmh.25-0226

**Published:** 2026-03-17

**Authors:** Maria Florinda João, Andrés Aranda-Díaz, Faith De Amaral, Takalani I. Makhanthisa, Sonja B. Lauterbach, Mukosha Chisenga, Brighton Mangena, Paulo Maquina, Isobel Routledge, Chadwick Sikaala, John Chimumbwa, Domingos Jandondo, José Franco Martins, Jaishree Raman, Jennifer L. Smith, Pedro Rafael Dimbu

**Affiliations:** ^1^ Programa Nacional do Controlo da Malária, Luanda, Angola;; ^2^ EPPIcenter Research Program, Department of Medicine, University of California, San Francisco, California, USA;; ^3^ Malaria Elimination Initiative, Global Health Group, University of California, San Francisco, California, USA;; ^4^ ISGlobal Barcelona, Spain;; ^5^ Laboratory for Antimalarial Resistance Monitoring and Malaria Operational Research, Centre of Emerging Zoonotic and Parasitic Diseases, National Institute for Communicable Diseases, Johannesburg, South Africa;; ^6^ SADC Malaria Elimination Eight Secretariat, Windhoek, Namibia;; ^7^ Instituto Nacional de Investigação em Saúde, Luanda, Angola;; ^8^ Wits Research Institute for Malaria, University of Witwatersrand, Johannesburg, South Africa;; ^9^ University of Pretoria Institute for Sustainable Malaria Control, University of Pretoria, Pretoria, South Africa;; ^10^ Department of Epidemiology & Biostatistics, University of California, San Francisco, California, USA

## Abstract

*Plasmodium falciparum* malaria remains a leading cause of mortality in Angola, with emerging antimalarial resistance threatening treatment and prevention strategies. Efficacy of artemether–lumefantrine has been reported below 90% in two provinces, underscoring the need for routine resistance surveillance. This study aimed to provide a geographically comprehensive and up-to-date overview of antimalarial drug resistance markers in Angola. Between March and July 2023, dried blood spots and demographic data were collected from *P*. *falciparum*-positive participants at 14 health facilities across 7 provinces. Multiplexed amplicon sequencing was used to characterize single nucleotide polymorphisms in 12 genes linked with resistance, estimate allele frequencies, and detect coinfecting non-falciparum *Plasmodium* species. Sequence data from 820 samples revealed significant geographic variation in resistance markers. In the southeast, artemisinin partial resistance markers (*k13* P574L, P441L) were detected at very low prevalence (<0.1%), whereas the quintuple *dhps*/*dhfr* haplotype, linked to sulfadoxine-pyrimethamine resistance, was very prevalent (>35% of samples). In the northwest, the sextuple *dhps*/*dhfr* haplotype, a marker of higher sulfadoxine-pyrimethamine resistance, was most prevalent in the Zaire province (14.2%). The chloroquine resistance marker *crt* C72/V73/M74I/N75E/K76T (CVIET) haplotype had a national prevalence of 17.7%, detected in over 48% of samples from the northern sites. The *mdr1* N86 genotype, linked to reduced lumefantrine susceptibility, was detected in 99.2% of samples. Coinfections of *P*. *falciparum* and non-falciparum species were rare, with no *Plasmodium vivax* coinfections detected. These findings highlight the need for continued monitoring to safeguard treatment efficacy, reinforcing the importance of molecular surveillance in malaria control strategies.

## INTRODUCTION

Angola is the seventh-most malaria-burdened country, globally, reporting 10,488,709 cases and 10,089 deaths in 2023 according to unpublished data from the Ministry of Health’s DHIS2 system.^1^ Malaria control relies on vector control, such as long-lasting insecticide-treated nets, indoor residual spraying, fumigation, and larviciding, together with case management strategies to ensure rapid and effective diagnosis and treatment of malaria infections.^2^ Preventive treatments for at-risk populations, including seasonal chemoprevention and intermittent preventive treatment in pregnancy (IPTp), are supported by efforts to promote community engagement and education of local leaders. However, operational and biological challenges, particularly the emergence of antimalarial drug resistance, threaten malaria control efforts.^3^

In Angola, chloroquine was used to treat uncomplicated malaria until the early 2000s, when confirmed chloroquine resistance prompted a transition to amodiaquine or sulfadoxine–pyrimethamine (SP).^4^^,^^5^ Increasing levels of SP resistance led to the adoption of artemisinin-based combination therapies (ACTs), the World Health Organization (WHO)-recommended first-line treatment, in 2006, with implementation completed by 2008.^4^^,^^6^ Artemisinin partial resistance (ART-R), characterized by delayed parasite clearance, is widespread in Southeast Asia and has recently emerged in East and the Horn of Africa.^7^^–^^10^

Since 2013, four of five therapeutic efficacy studies conducted in Angola have reported artemether–lumefantrine (AL) efficacy below 90%, the WHO threshold at which further investigation and potential revisions to national treatment guidelines are recommended.^11^^–^^15^ These reports of reduced AL efficacy are localized primarily to the northern province of Zaire. The other ACTs used in-country, namely artesunate–amodiaquine (ASAQ), dihydroartemisinin–piperaquine (DHAP), and artesunate–pyronaridine, have remained effective.^11^^–^^15^ No studies evaluating the chemopreventive efficacy of SP have been conducted in Angola. However, reports from studies elsewhere suggest that SP-based IPTp continues to provide clinical benefits, even in the presence of resistant strains.^16^^–^^18^

Molecular surveillance of mutations associated with antimalarial resistance complements therapeutic efficacy and chemopreventive efficacy studies, serving as an early warning mechanism and guiding efficacy assessments.^19^ ART-R is associated with single-nucleotide polymorphisms (SNPs) in the propeller domain of the Kelch13 (K13) protein, encoded by the *k13* gene.^20^ These SNPs are classified based on available clinical or laboratory evidence: validated markers require both types of evidence, whereas candidate markers are supported by only one.^21^ Over the past decade, the validated markers C469Y, R561H, R622I, and A675V, along with the candidate markers P441L and C469F, have been increasingly reported in Ethiopia, Eritrea, Tanzania, Uganda, and Rwanda.^9^^,^^10^^,^^22^^–^^25^

Chloroquine resistance is associated with mutations in the chloroquine resistance transporter (*crt*) gene, particularly the K76T mutation within the *crt* C72/V73/M74I/N75E/K76T (CVIET) haplotype, which was dominant in Africa.^26^^–^^28^ Chloroquine resistance is further modulated by additional mutations in the *crt* and multidrug resistance1 (*mdr1*) genes. Mutations in these two genes are also associated with resistance or reduced susceptibility to piperaquine, amodiaquine, and lumefantrine.^29^^,^^30^ Copy number variations in the *plasmepsin 2* and *3* genes are associated with piperaquine resistance, whereas duplications in *mdr1 gene* are associated with mefloquine resistance. Resistance to SP is driven by the accumulation of SNPs in the dihydropteroate synthase (*dhps*) and dihydrofolate reductase (*dhfr*) genes.^31^

Studies in Angola over the past decade have reported a very low prevalence of *k13* mutations, with no validated or candidate ART-R markers detected.^32^^–^^36^ The chloroquine resistance *crt* K76T marker and the *mdr1* N86 genotype, which is linked to reduced lumefantrine susceptibility, were frequently identified; however, mutations in the *dhps* and *dhfr* genes associated with high SP resistance have remained rare.^14^^,^^32^^,^^34^^,^^36^ The rapid emergence of ART-R markers in East and the Horn of Africa underscores the need for routine, geographically representative molecular surveillance to inform timely policies.

To provide a geographically comprehensive and up-to-date overview of antimalarial drug resistance markers in Angola, the Genomics for Malaria in the Elimination 8 (GenE8) initiative, a regional effort aimed at strengthening malaria genomic surveillance in five Southern African countries (Angola, Eswatini, Namibia, South Africa, and Zambia), conducted molecular malaria surveillance, generating targeted amplicon deep sequencing from seven provinces of Angola. Here we report the prevalence of molecular markers associated with resistance to the most widely used antimalarials, based on malaria-positive samples collected between March and July 2023.

## MATERIALS AND METHODS

### Study site.

Angola comprised 18 provinces at the time of the study and shares borders with the Democratic Republic of the Congo, Zambia, and Namibia (Figure 1). Malaria prevalence, based on rapid diagnostic tests (RDTs), is 17% among children aged 6 to 59 months.^37^^,^^38^ Because transmission intensity varies significantly across the country and within provinces, malaria transmission is stratified at the municipal level (Figure 1) into four categories: very high (incidence >1,000 cases per 1,000 inhabitants, prevalence >50%), high (incidence 500–1,000/1,000, prevalence between 30 and 50%), moderate (incidence 300–500/1,000 inhabitants, prevalence 10–30%), and low (incidence <300/1,000, prevalence <10%).^39^ Transmission occurs year-round, with peaks between March and May. The predominant parasite species is *Plasmodium falciparum*, although *Plasmodium malariae*, *Plasmodium ovale*, and *Plasmodium vivax* are occasionally reported.^40^^,^^41^ The primary malaria vectors include *Anopheles gambiae* s.l., *An*. *arabiensis*, and *An*. *funestus* s.l.^39^ This study was conducted in eight provinces (Figure 1) where 100% of the population, estimated at 10.5 million (30.9% of the total population), is at risk of contracting malaria. The healthcare system in these 8 provinces comprises 897 health facilities that serve as centers that diagnose and treat malaria. Diagnosis is conducted at the point of care using *P*. *falciparum* histidine rich protein 2 (HRP2)/lactate dehydrogenase (LDH)-based RDTs or microscopy. All patients with uncomplicated malaria receive one of three first-line ACTs (AL, ASAQ, or DHAP). Severe malaria cases are treated with intravenous artesunate, intramuscular artemether, or injectable quinine, followed by a full course of an ACT. SP is used as IPTp in all health facilities with antenatal care services.

**Figure 1.**
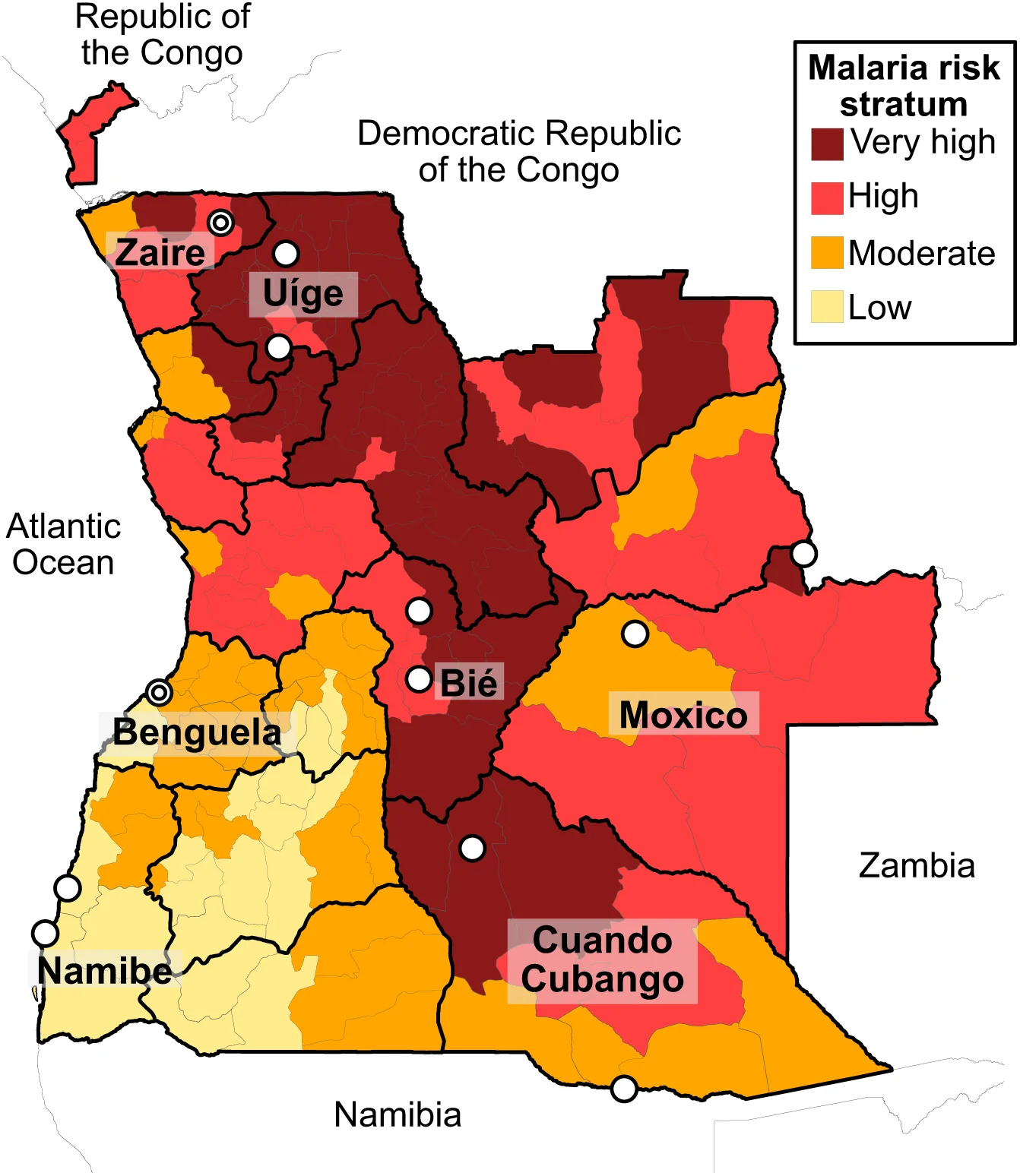
Angola malaria risk stratifications.^2^ Risk strata are shown in different colors (see Study Site section for definitions). Health facilities where samples were collected are indicated with circles, with overlapping facilities represented by two concentric circles. Health facilities from Lunda Sul were excluded from the analysis and are not shown.

### Study design and field procedures.

This cross-sectional, health facility-based study was conducted as part of the Regional GenE8 project and was integrated into Angola’s national surveillance system. Eight provinces were selected, including two health facilities each, to ensure geographic and transmission-strata representativeness.

Zaire, Lunda Sul, and Benguela provinces each host two therapeutic efficacy study (TES) sites, which were included in this study. The remaining 10 health facilities were selected from other 5 provinces (Bié, Cuando Cubango, Moxico, Namibe, and Uíge), ensuring representation across all four transmission strata while considering accessibility and patient flow. The study aimed to successfully sequence at least 50 RDT-positive samples from each of the 16 health facilities. To achieve this, 200 RDT-positive patients were targeted for dried blood spot (DBS) sample and demographic data collection. The total sequenced target sample size (*N* = 800) was designed to: 1) estimate a 5% prevalence of a specific molecular marker with 1.85% precision at 95% confidence, and 2) achieve 90% power to detect a ≥2% prevalence if the true prevalence is 4.16% using a one-sided binomial test (α = 0.05, design effect [Deff] = 1.5). The targeted sample size was inflated to 200 RDT-positive patients per facility to ensure high-quality sequencing of the minimum sample size and retain additional samples in a regional biobank for follow-up investigations. Study activities took place from March to July 2023 during the malaria transmission season, with recruitment staggered across health facilities. All symptomatic patients older than two years who tested positive for *P*. *falciparum* malaria by an HRP2/LDH-based RDT (SD Bioline P.f./P.v., Gyeonggi-do, Republic of Korea) were invited to participate in the study. Patients with a history of fever in the previous two weeks or severe malaria were excluded. In most facilities, sample and data collection targets were met within two weeks. However, the collection period was extended to three months in Hospital Geral de Benguela (Benguela province) and Hospital Municipal do Calai (bordering Namibia in Cuando Cubango province) because of low case numbers.

Before the study enrollment, written informed consent was obtained as described in the Ethical Considerations section. Recruited participants were interviewed at the point of care using paper forms to collect demographic data, clinical symptoms, travel history, and occupation. Finger-prick DBS samples were collected on filter paper strips (Cytiva Whatmann 3MM Chr, Wilmington, DE), labeled with unique barcodes linking survey data, air-dried, and stored individually in sealed bags with desiccant. Labeled samples were sent to the National Institutes of Health Research in Luanda, Angola, before being shipped to the regional sequencing hub at the National Institute for Communicable Diseases in South Africa. Survey forms were stored at the National Malaria Control Program, where the demographic data were digitized using CommCare software on encrypted Android tablets. Questionnaires from the two health facilities in Lunda Sul province were misplaced; therefore, data from the province were excluded from all analyses.

### Sample processing and genomic assay.

DNA was extracted from at least 90 samples per health facility (one 6-mm DBS disc per sample) using the Chelex-Tween 20 method.^42^ Extractions were performed in batches of 96 samples, each including 3 positive controls (infected blood and 2 cultured strains mixed with human blood and spotted on filter paper) and 3 negative controls (uninfected blood on filter paper, clean filter paper, and no input control). Parasite densities were estimated using the varATS quantitative polymerase chain reaction (qPCR) assay.^43^ A minimum of 70 *P*. *falciparum*-positive samples per health facility with >1,000 parasites/*µ*L of blood were randomly selected for sequencing. If this target could not be achieved, samples with parasitemia as low as 10 parasites/*µ*L of blood were included.

Amplicon sequencing libraries were prepared using the D1.1, R1.2, and R2.1 pools of the Multiplexed Amplicons for Drugs, Diagnostics, Diversity, and Differentiation using High-Throughput Targeted Resequencing (MAD^4^HatTeR) panel. Libraries were prepared in 96-well plates and included a positive control (DNA extracted from cultured strain control) and 4 no-template controls in each plate. Libraries were sequenced on a NextSeq 2000 instrument using 150-cycle paired-end reads.^44^ A custom bioinformatics pipeline was used to infer alleles at 241 targets.^44^ This report includes sequences from the following genes: *k13*, *crt*, *mdr1*, multidrug resistance 2 (*mdr2*), *dhps*, *dhfr*, *coronin*, apicoplast ribosomal protein S10 (*arps10*), putative phosphoinositide-binding protein (*pib7*), ferrodoxin (*fd*), exonuclease (*exo*), and PF3D7_1322700. Lactate dehydrogenase (*ldh*) gene target sequences in *P*. *falciparum*, *P*. *vivax*, *P*. *malariae*, *P*. *ovale* and *P*. *knowlesi* were used to identify the presence of *Plasmodium* species in the samples. SNPs in *ldh* targets were used to distinguish *P*. *ovale curtisi* from *P*. *ovale wallikeri*. Microhaplotypes are reported for SNPs within the same amplicon. Modifications to the original protocol and bioinformatics pipeline are described in Eloff et al.^45^

### Data cleaning, analysis, and visualization.

Data cleaning, analysis, and visualization were conducted in R (version 4.3.1). Samples with <10,000 reads, targets with <50 reads, and SNPs with within-sample allele frequency (WSAF) <0.01 or with ≤10 reads were excluded from the analysis. SNPs observed in only one sample with WSAF <1, and SNPs observed in multiple samples but exclusively in a single sequencing run, were also excluded. Cross-sample contaminations were identified in positive controls with unexpected alleles compared with reference genomes and in negative controls with reads. Batches with more than one control showing contamination (>100 reads in >10 targets) were excluded.

Non-*falciparum* targets were excluded from sequencing depth summaries. Otherwise, all resulting data were analyzed, including samples with partial SNP coverage, leading to variable total sample numbers for each assessed SNP. Samples were considered successfully sequenced if a genotype was obtained for all 59 SNPs evaluated in the *k13* gene. Multilocus haplotypes for polymorphisms in 5 independent targets in *dhps* and *dhfr* genes (*dhps* 436, 437, 540, 581, and 613; and *dhfr* 51, 59, 108, and 164), 3 independent targets in the *mdr1* gene (amino acids 86, 184, and 1,246), and 3 independent targets in the *crt* gene (*crt* 72–76, 220, 356) were inferred for each sample by combining all individual haplotypes. Haplotypes were inferred for samples with either unmixed genotypes at all targets or with a single target showing a mixed genotype, assuming such samples carried the two haplotypes accounting for the mixture. Haplotype classification for *dhps* (wild type, <2 mutations, double or triple), *dhfr* (wild type, <3 mutations, triple or quadruple), and *dhps*/*dhfr* (wild type, <5 mutations, quintuple or sextuple) are detailed in Supplemental Table 1. Samples with mixed genotypes in more than two amplicons were classified as indeterminate.

To assess data patterns and identify potential issues with sampling and library preparation, sample similarity was estimated using the root-mean-square error of WSAF for highly diverse loci (pool D1.1 targets), followed by Density-Based Spatial Clustering of Applications with Noise with ε = 0.2. Samples with clonality >2 and nearly identical WSAF across all loci, suggesting contamination or sample duplication, were excluded from the analysis.

## STATISTICAL ANALYSES

Descriptive statistics for gender, age group (<5, 5–14, 15–24, 25–39, 40–59, >59 years), occupation (including the self-defined occupation category “minor”), overnight travel in the past two months, and country of residence were calculated. To facilitate statistical analysis, some variables were reclassified: occupations in agriculture, herding, and fishing were combined into a single occupational category (agricultural workers); individuals aged 25 years or older were grouped into a single category; and travel reports (within two months before sample collection) were categorized as international or domestic. Differences between successfully sequenced samples and qPCR-positive samples, as well as between provinces, were assessed using χ^2^-tests.

The proportion of samples carrying a mutation or haplotype was calculated as the number of mixed and pure mutant genotypes divided by the total number of genotyped infections. Proportions of the *dhps* and *dhfr* haplotypes were determined using the haplotype with the most mutations observed in each sample (Supplemental Table 1). The prevalence of infections carrying a genotype at the national level was adjusted considering clustered sampling, and survey weights were applied to adjust for unequal sampling probabilities across provinces, health facilities, and participants using the R package survey (v.4.4.2; https://cran.r-project.org/web/packages/survey/index.html). The beta method was used to obtain 95% confidence intervals.

Univariate logistic regression was performed using generalized linear mixed models to assess associations between the presence of markers of interest (binary outcome) and key categorical variables, including age group, gender, and occupation, incorporating a random intercept for each health facility and fixed effects for all other variables. Multivariable logistic regression was subsequently conducted. Because of the co-occurrence between certain occupational categories and age groups (e.g., agricultural workers predominantly in older groups), separate mixed-effects models were fitted: one including gender and age, and another including gender and occupation. Analyses were restricted to mutations and variables with a sufficient sample size to achieve model convergence.

The complexity of infection (COI) for each sample and population allele frequencies were jointly estimated for each municipality and province using Multiplicity Of Infection and allele frequency REcovery from noisy polyallelic data (MOIRE, v3.4.0; https://github.com/EPPIcenter/moire), which implements a Markov chain Monte Carlo-based approach to jointly infer sample COI and within host relatedness, and population allele frequencies, using polyallelic genomic data.^46^ By modeling the genetic data and accounting for experimental errors, MOIRE provides probabilistic estimates. Microhaplotypes in all highly diverse *P. falciparum* targets (primer pool D1.1), as well as SNPs or microhaplotypes in drug resistance targets of interest, were used to infer COI and allele frequencies. The percentage of polyclonal infections was estimated as the mean of individual probabilities of polyclonality.

Non-*falciparum* species were considered present if the combined reads across all *ldh* targets exceeded 100, with each species contributing more than 1% of the total *ldh* target reads.

### Data availability.

A dataset containing all individual genotypes, COI, and probabilities of polyclonality is available online (DOI:10.5281/zenodo.18697369).

## RESULTS

DNA was extracted and parasite density quantified from 1,479 samples. To reach the target sample size for sequencing, libraries were prepared for 856 samples. Because of potential cross-contamination, 15 sequenced samples were excluded from further analyses. At least one SNP genotype was obtained for 97.5% (820/841) of the samples from the 14 health facilities, and 722 (85.9%) were successfully sequenced (i.e., genotyped for all 59 *k13* SNPs). Sequencing success rates varied across provinces (Table 1), with Zaire having the highest number of successfully sequenced samples and Namibe having the lowest number.

**Table 1:** Demographics of study participants with successfully sequenced samples

Characteristic	Overall	Benguela	Bié	Cuando Cubango	Moxico	Namibe	Uíge	Zaire
*N*	722	113	118	93	105	57	105	131
Health facilities	14	2	2	2	2	2	2	2
Age								
<5 years	168 (23%)	23 (20%)	37 (31%)	18 (19%)	33 (31%)	9 (16%)	20 (19%)	28 (21%)
5–14 years	287 (40%)	68 (60%)	43 (36%)	35 (38%)	33 (31%)	22 (39%)	33 (31%)	53 (40%)
15–24 years	156 (22%)	11 (9.7%)	12 (10%)	30 (32%)	23 (22%)	19 (33%)	31 (30%)	30 (23%)
≥25 years	111 (15%)	11 (9.7%)	26 (22%)	10 (11%)	16 (15%)	7 (12%)	21 (20%)	20 (15%)
Gender								
Female	384 (53%)	63 (56%)	66 (56%)	43 (46%)	63 (60%)	26 (46%)	62 (59%)	61 (47%)
Male	338 (47%)	50 (44%)	52 (44%)	50 (54%)	42 (40%)	31 (54%)	43 (41%)	70 (53%)
Occupation								
Agriculture	66 (9.2%)	0 (0%)	17 (14%)	8 (8.6%)	7 (6.7%)	0 (0%)	28 (27%)	6 (4.6%)
Student	297 (41%)	55 (49%)	27 (23%)	38 (41%)	38 (36%)	27 (50%)	35 (34%)	77 (59%)
Other	68 (9.5%)	11 (9.7%)	12 (10%)	7 (7.5%)	11 (10%)	13 (24%)	5 (4.8%)	9 (6.9%)
Minor	257 (36%)	44 (39%)	57 (48%)	31 (33%)	43 (41%)	11 (20%)	35 (34%)	36 (27%)
Unemployed	30 (4.2%)	3 (2.7%)	5 (4.2%)	9 (9.7%)	6 (5.7%)	3 (5.6%)	1 (1.0%)	3 (2.3%)
Unknown	4	0	0	0	0	3	1	0
Travel history								
Domestic	49 (6.8%)	7 (6.2%)	16 (14%)	19 (20%)	6 (5.7%)	0 (0%)	1 (1.0%)	0 (0%)
International	1 (0.1%)	0 (0%)	0 (0%)	0 (0%)	0 (0%)	1 (1.8%)	0 (0%)	0 (0%)
No travel reported	672 (93%)	106 (94%)	102 (86%)	74 (80%)	99 (94%)	56 (98%)	104 (99%)	131 (100%)

The median sequencing depth per sample was 617,873 reads (interquartile range [IQR]: 164,192–1,333,110), with a median depth per target of 1,278 reads (IQR: 266–4,100). A median of 223 of 238 targets (IQR: 207–226) had more than 100 reads. SNPs with the lowest genotyping success rates included *crt* 145, *crt* 218/220, and *mdr1* 86 (genotyped in 651, 226, and 649 samples, respectively). More than two-thirds of the analyzed samples (68.4%) were polyclonal, with a population mean COI of 2.94 (95% confidence interval [CI]: 2.91–3.01, *N* = 820). Mixed genotypes in at least one drug resistance marker were detected in 58% of samples. Coinfecting non-*falciparum* species (*P. malariae*, *P. ovale curtisi*, and *P. ovale wallikeri*) were rare, collectively detected in 5.6% of samples (Supplemental Table 2). No samples containing *P. vivax* were identified.

No significant differences in province, gender, or travel history were observed between individuals with qPCR-positive samples and those with successful sequenced samples (Supplemental Table 3). However, younger age groups and members of specific occupational categories (“students” and “minors”) were overrepresented among successfully sequenced samples compared with qPCR-positive samples. The majority of sequenced samples came from students (41%) or minors (36%), with only 15% of participants over the age of 24 years (Table 1). Gender distribution was balanced (53% females), and all participants were Angolan. Travel was reported by 6.9% of participants, with just one case of international travel recorded.

### Mutations in the crt gene.

Six nonsynonymous mutations were detected in the *crt* gene (Table 2). The chloroquine-resistant *crt* CVIET microhaplotype had an adjusted national prevalence of 17.7% (CI 3.5–44.8%, *N* = 820, Deff = 39.2, intraclass correlation coefficient [ICC] = 0.66; Figure 2A, Supplemental Table 4) and was most abundant in the provinces of Uíge (58.9%, *N* = 124) and Zaire (48.5%, *N* = 134, Figure 2B, Supplemental Tables 5 and 6), where its allele frequency exceeded 21% (Supplemental Table 7). In contrast, its abundance was low in the southeastern provinces of Cuando Cubango and Moxico (<2%, *N* = 114 and 116). Similar adjusted national prevalences were observed for the *crt* A220S (10%, CI 1.9–27.5%, *N* = 226, Deff = 6.15, ICC = 0.34) and I356T (12.2%, CI 2.0–34.5%, *N* = 770, Deff = 28.9, ICC = 0.52, Figure 2A) mutations, which were also more frequent in the northern provinces (Figure 2B, Supplemental Tables 5–7). The *crt* A220S mutation was generally but not exclusively found in samples with the CVIET microhaplotype and *crt* I356T mutation (Supplemental Table 8). The *crt* I356T was always found in a haplotype with the CVIET microhaplotype and *crt* A220S.

**Table 2:** Summary of nonsynonymous mutations

Gene	Observed in >0.5% of Samples	Observed in ≤0.5% of Samples
*k13*		
Validated ART-R markers		P574L
Candidate ART-R markers		P441L
No evidence of ART-R	A578S, Q613E	P667A
*crt*	M74I, N75E, K76T, A220S, I356T	F48L
*dhfr*	N51I, C59R, S108N	–
*dhps*	S436A, A437G, K540E, A581G, A613S	I431V
*mdr1*	N86Y, F184Y	D1246Y
*mdr2*	I492V	–
PF3D7_1322700	T236I	–

Markers of partial artemisinin resistance (ART-R) are categorized as validated or candidate, based on the available clinical and laboratory evidence. Validated markers have both types of evidence, whereas candidate markers are supported by only one, by following the current World Health Organization classification.^47^

**Figure 2.**
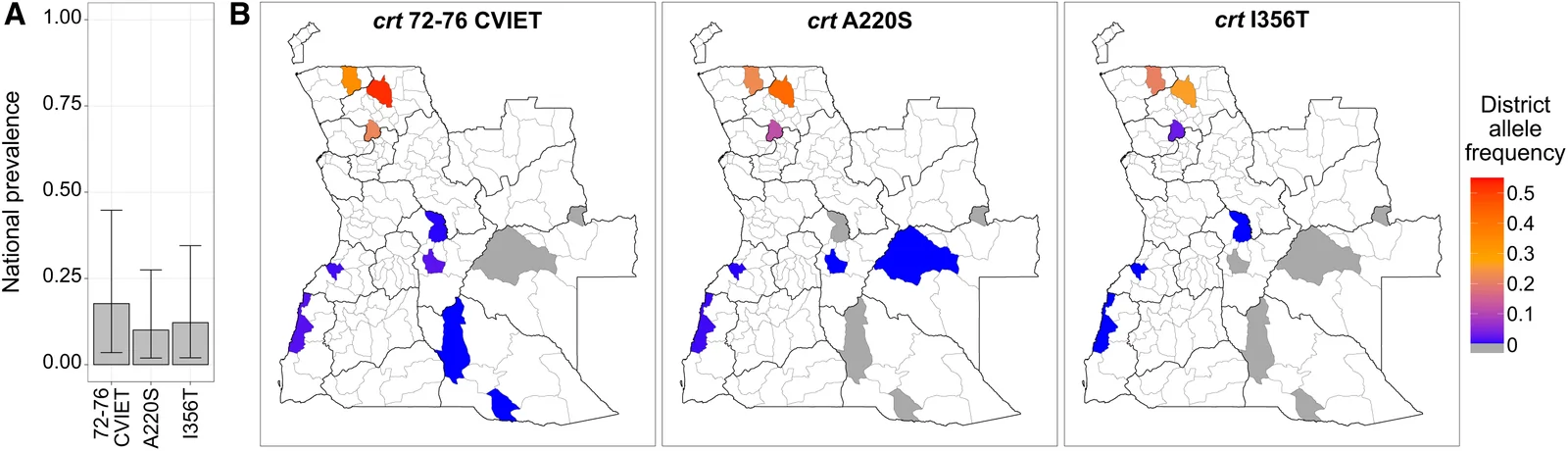
Mutations detected in the chloroquine resistance transporter (*crt*) gene. (**A**) National adjusted prevalence of infections carrying a *crt* genotype. (**B**) Estimated allele frequencies at the municipal level for the detected *crt* genotypes.

Participants older than 24 years old had significantly higher odds of carrying the CVIET microhaplotype (odds ratio [OR]: 2.66; CI: 1.31–5.41; *P* = 0.007, Supplemental Tables 9–11) and the *crt* I356T mutation (OR: 2.36; CI: 1.04–5.34; *P* = 0.04) compared with children under the age of 5 years. Similarly, agricultural workers were significantly more likely to carry the CVIET microhaplotype (OR: 2.65; CI: 1.26–5.58; *P* = 0.01) and the *crt* I356T mutation (OR: 2.45; CI: 1.05–5.72; *P* = 0.038) compared with minors. No significant associations were observed for gender and other occupational groups. Of the 156 samples carrying the CVIET microhaplotype, only 4 belonged to participants who reported domestic travel, and in two cases, travel occurred within the same district where they were sampled. Sample size was insufficient to assess associations between the *crt* A220S mutation and demographic factors.

### Mutations in the *dhps* and *dhfr* genotypes.

A *dhps* haplotype could be inferred for 95.8% (758 of 791) of the samples with genotypes available at all 4 *dhps* SNPs, and a *dhfr* haplotype was inferred for all 800 samples genotyped for all 3 *dhfr* SNPs (Supplemental Tables 12 and 13). The *dhps* double mutant haplotype (S436A or A437G, plus K540E), associated with SP resistance, was present in 41.1% of the samples, whereas 3.0% carried a *dhps* triple mutant haplotype (A581G or A613S in addition to the double mutant haplotype) linked to higher SP resistance levels. The *dhps* triple mutant haplotypes were particularly prevalent in northern Zaire province (Figure 3A). The *dhfr* triple mutant haplotype (N51I, C59R, and S108N), linked to pyrimethamine resistance, was found in 87.4% of samples. Because of polyclonality and the presence of mixed genotypes, a full *dhps*/*dhfr* haplotype could be inferred for only 644 of 790 samples (81.5%) with complete genotypes for both genes (Supplemental Table 14). Among these, 28.8% carried the quintuple mutant haplotype (*dhps* double mutant plus *dhfr* triple mutant), and 2.64% carried the sextuple mutant haplotype (*dhps* triple mutant plus *dhfr* triple mutant, Figure 3A).

**Figure 3.**
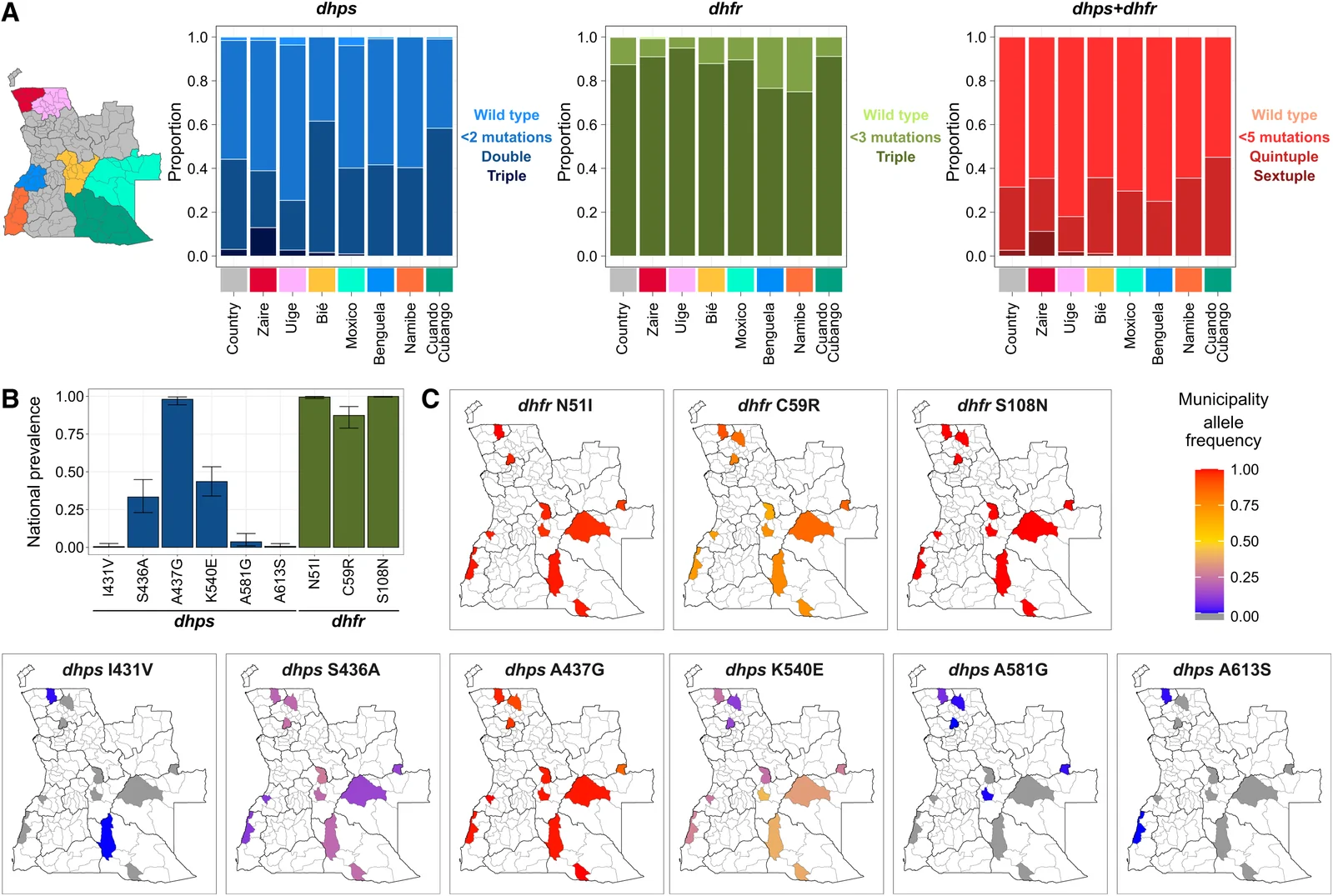
Mutations detected in the dihydropteroate synthase (*dhps*) and dihydrofolate reductase (*dhfr*) genes. (**A**) Proportion of samples carrying a haplotype for *dhps*, *dhfr*, or *dhps+dhfr* at the provincial level, excluding undetermined samples. (**B**) National adjusted prevalence of infections carrying a genotype in the *dhps* or *dhfr* gene. (**C**) Estimated allele frequencies at the municipal level for the same markers.

The *dhps* A437G mutation had an adjusted national prevalence of 98.1% (CI: 94.4–99.6%, *N* = 804, Deff = 4.01, ICC = 0.05, Figure 3B, Supplemental Table 4). The *dhps* S436A mutation had an adjusted national prevalence of 33.3% (CI: 23.1–44.8%, *N* = 804, Deff = 7.67, ICC = 0.11) occurring in a microhaplotype with *dhps* A437G in 278 of 287 samples (96.9%) carrying it. The *dhps* I431V mutation was detected in four samples from the Zaire province, with an adjusted national prevalence of 0.4% (CI: 0.01–2.5%, *N* = 804, Deff = 2.8, ICC = 0.03), always in a microhaplotype with *dhps* S436A and A437G. The *dhps* K540E mutation, the hallmark of the *dhps* double haplotype, had an adjusted national prevalence of 43.4% (CI: 33.9–53.4%, *N* = 761, Deff = 5.27, ICC = 0.08, Figure 3B). This mutation was less frequent in the northern provinces of Zaire and Uíge, where municipal allele frequencies were below 25%, whereas in southern Cuando Cubango, they exceeded 38% (Figure 3C, Supplemental Tables 5–7).

The *dhps* triple mutant haplotype SNPs, *dhps* A581G, and A613S had a low prevalence and were primarily found in the northern provinces (Figure 3B and C). The *dhps* A581G mutation was detected in 14.2% of samples from Zaire, with an adjusted national prevalence of 3.6% (CI: 0.9–9.1%, *N* = 808, Deff = 5.7, ICC = 0.08). It was present in a microhaplotype with *dhps* K540E in 23 of 27 samples (85.2%). The *dhps* A613S mutation was identified in four samples from Zaire in a haplotype with *dhps* K540E and A581G and in one sample from Namibe in a haplotype with *dhps* K540E and wild-type A581, with an adjusted national prevalence of 0.6% (CI: 0.1–2.4%, *N* = 914, Deff = 2.1, ICC = 0.02).

Mutations in *dhfr* gene had the following adjusted national prevalence: N51I 99.5% (CI: 9.86–99.9%, *N* = 768, Deff = 1.1, ICC = 0.01), C59R 87.3% (CI: 79.0–93.2%, *N* = 766, Deff = 6.0, ICC = 0.09), and S108N 99.9% (CI: 99.5–100.0%, *N* = 820, Deff = 0.58, ICC = 0.00, Figure 3B). The C59R mutation was less frequent in Namibe and Benguela provinces, where municipal allele frequencies were ≤70% (Figure 3C, Supplemental Tables 5–7). The *dhfr* I164L mutation, associated with higher pyrimethamine resistance levels, was not detected.

Participants older than over 24 years old had significantly higher odds of carrying the *dhps* K540E mutation (OR: 1.85; CI: 1.14–2.99; *P* = 0.013, Supplemental Tables 9 and 10) compared with children under the age of 5 years. Similarly, agricultural workers were significantly more likely to carry the *dhps* K540E mutation (OR: 1.95; CI: 1.11–3.45; *P* = 0.021, Supplemental Tables 9 and 11) compared with those aged less than 5 years and minors. Males were more likely to carry the *dhps* S436A mutation than females, with an odds ratio of 1.43 (CI: 1.05–1.94, *P* = 0.025). No significant associations were found between demographic factors and the presence of *dhps* A581G.

### Mutations in the *k13* gene.

Although several nonsynonymous *k13* mutations were detected, only two were found in >0.5% of samples (Table 2, Figure 4, Supplemental Tables 4–6). *k13* A578S was detected in Bié (2.3%, *N* = 132), Moxico (1.7%, *N* = 116), and Benguela (0.8%, *N* = 125), with an adjusted national prevalence of 0.7% (CI: 0.1–2.3%, *N* = 810, Deff = 1.9, ICC = 0.02). *k13* Q613E was detected in Cuando Cubango (4.4%, *N* = 113), Benguela (2.4%, *N* = 125), Namibe (1.5%, *N* = 68), and Bié (0.8%, *N* = 132), with an adjusted national prevalence of 1.5% (CI: 0.2–5.2%, *N* = 810, Deff = 4.2, ICC = 0.06).

**Figure 4.**
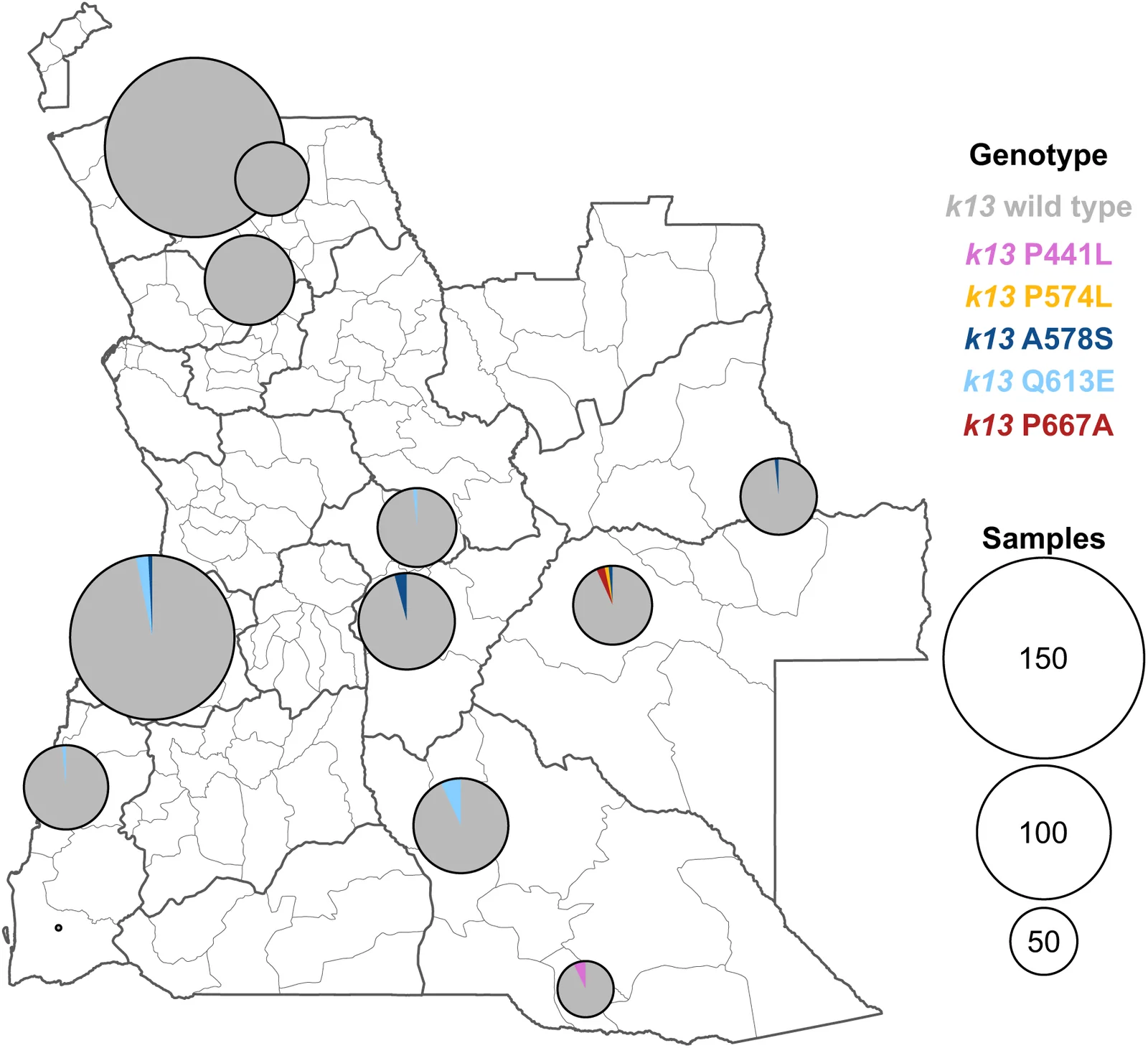
Nonsynonymous mutations in *k13*. Proportion of samples carrying a nonsynonymous mutation in *k13* at the municipal level.

Two markers of ART-R were observed: one (1.7%) sample from Luena municipality, Moxico province, carried the validated marker *k13* P574L, whereas three samples (7.3%) from Calai municipality, Cuando Cubango province, carried the candidate marker *k13* P441L. The *k13* P667A mutation was found in two (4.1%) samples in Luena municipality, Moxico province. Synonymous mutations were detected but at low proportions, including *k13* C494C, below 1%.

No significant association was found between the presence of non-synonymous *k13* mutations and age or gender (Supplemental Tables 8 and 9). No participant with a nonsynonymous *k13* mutation reported traveling outside their province.

### Mutations in other genes.

Nearly all samples (644 of 649, or 99.2%) carried the wild-type *mdr1* N86 genotype. The adjusted national prevalence of *mdr1* N86Y was 0.7% (CI: 0.2–1.9%, Deff = 1.0, ICC = 0.00), with the highest proportion of samples in the Zaire province, where 4.69% of samples carried the mutation (*N* = 128, Supplemental Tables 5 and 6). In contrast, *mdr1* Y184F had an adjusted national prevalence of 53.2% (CI: 48.3–58.1%, *N* = 788, Deff = 1.42, ICC = 0.01). Three samples, one each from Uíge, Cuando Cubango, and Moxico, carried *mdr1* D1246Y. No mutations were detected at positions 1,034 or 1,042. Among the 642 samples with complete genotypes at *mdr1* codons 86, 184, and 1,246 whose haplotype could be inferred, 50.8% carried the NFD haplotype and 79.1% carried the NYD haplotype (Supplemental Table 15). No associations between the *mdr1* Y184F mutation and age, gender, or occupation were observed (Supplemental Tables 9–11).

The PF3D7_1322700 T366I mutation had an adjusted national prevalence of 1.3% (CI: 0.3–3.6%, *N* = 658, Deff = 1.89, ICC = 0.02), with the highest prevalence in Benguela, where 4.9% (*N* = 103) of samples carried it (Supplemental Tables 5 and 6). Finally, the *mdr2* I492V mutation had an adjusted national prevalence of 61.1% (CI: 53.6–68.2%, *N* = 739, Deff = 3.04, ICC = 0.04) and was significantly less likely to be carried by participants aged 5–14 years compared with children under the age of 5 years (OR: 0.65; CI: 0.43–0.96; *P* = 0.031, Supplemental Tables 9–11).

## DISCUSSION

In this study, we assessed the national prevalence of antimalarial drug resistance markers using samples collected across 7 of 18 Angola provinces using amplicon deep sequencing. Resistance makers prevalence was highly heterogeneous, with the *crt* K76T mutation associated with chloroquine resistance, prevalent in the northwestern provinces (Uíge and Zaire), whereas *dhps* K540E, linked to SP resistance, was most common in the eastern provinces. Although the *dhps* K540E allele was relatively rare in the northwestern provinces, the *dhps* A581G and *dhps* A613S mutations, associated with higher levels of SP resistance when present in addition to the quintuple mutation, were more common in these provinces. The validated ART-R *k13* P574L marker and the candidate *k13* P441L marker were detected at very low prevalence in only two southeastern provinces (Cuando Cubango and Moxico). The *mdr1* N86 wild-type genotype, associated with reduced lumefantrine susceptibility, was detected in nearly all samples analyzed.^30^ Low-level *P*. *falciparum* coinfections with *P*. *malariae*, *P*. *ovale* curtisi, and *P*. *ovale* wallikeri were identified.

Chloroquine was removed from Angola’s national malaria treatment guidelines in 2006 and replaced with three ACTs (AL, ASAQ, and DHAP).^2^ The *crt* CVIET haplotype, observed at a national prevalence of 15.9%, is a strong predictor of chloroquine treatment failure but has a less clearly defined association with amodiaquine and quinine susceptibility.^26^^–^^28^^,^^48^ The *crt* CVIET haplotype and two additional *crt* mutations, *crt* A220S and *crt* I356T, which can confer even higher levels of chloroquine resistance in the presence of the *crt* CVIET haplotype, were detected at high frequencies in the northwestern provinces of Zaire and Uíge.^49^ These findings align with data from a 2018 study in Cabinda, a coastal exclave in northern Angola.^34^ Malaria molecular data from countries neighboring Angola are scarce, but two studies between 2013 and 2017 have shown that the CVIET haplotype, along with A220S and I356T mutations, are highly prevalent in regions of the Democratic Republic of Congo (DRC) proximal to Zaire and Uíge provinces.^50^^,^^51^ Although chloroquine is no longer formally used for malaria treatment in Angola, the DRC, or other nearby countries, the results from this study suggest selective pressure for these mutations persists. Parasite migration across borders may also contribute to their continued presence in the region. The SVMNT haplotype, linked to amodiaquine resistance, detected in Angola’s capital Luanda in 2007, was not detected in this study.^52^

Mutations in the *dhps* and *dhfr* genes associated with SP in vitro and in vivo resistance impact treatment efficacy and may also reduce the efficacy of SP in chemoprevention.^31^^,^^53^ Like the *crt* mutations, the prevalence of the *dhps* and *dhfr* mutations was heterogeneous. The *dhps* K540E mutation, a hallmark of the quintuple mutant haplotype and a strong predictor of SP treatment failure in East and southern Africa, was more prevalent in the eastern provinces, whereas sextuple mutant haplotypes were concentrated in the northwestern regions. The quintuple mutant haplotype has been linked to reduced SP efficacy for both treatment and perennial malaria chemoprevention in children.^53^^–^^56^ Additionally, seasonal malaria chemoprevention (SMC) has been shown to select for this haplotype.^57^^,^^58^ Furthermore, the *dhps* I431V-containing VAGKGS haplotype was rare and localized to the north of the country. This haplotype has previously been reported in West and Central Africa, and its effect on SP-based treatment or chemoprevention remains unknown.^59^^–^^61^

However, SP-based IPTp has been demonstrated to improve birth outcomes even in areas where the quintuple mutant haplotype is prevalent.^16^^–^^18^ Certain SNPs in the sextuple mutant haplotype, such as *dhps* A581G SNP found in 14.2% of samples from Zaire, may reduce the effectiveness of SP for IPTp.^62^^–^^65^ Despite these concerns, the WHO continues to recommend SP for IPTp and SP in combination with amodiaquine for SMC, citing insufficient evidence of the negative impact of these mutations on intervention efficacy.^66^ Predominantly found in the northwestern province of Zaire in this study, the *dhps* A581G mutation was detected at very low levels in regions of the DRC neighboring Zaire in 2013–2014, reaching nearly 10% prevalence in Cabinda in 2018 and was observed at 3.4% in southern Republic of Congo in 2021.^34^^,^^50^^,^^67^^–^^69^ This suggests an increase over the last decade or a highly heterogeneous distribution in the region. In contrast, the *dhps* K540E mutation was more prevalent in eastern provinces. This mutation was also twice as likely to be carried by agricultural workers compared with minors, resembling associations previously observed in minors in the DRC.^67^ Overall, these findings highlight the geographical variation in *dhps* and *dhfr* mutations and reinforce the need for continued surveillance; however, current IPTp and SMC policies remain appropriate and should be maintained while monitoring continues.

The rapid rise of mutations in the *k13* gene over the past decade has underlain the emergence of ART-R in Africa. The validated *k13* P574L marker, identified in a single sample from Moxico province, has also been detected at low frequencies in Western Zambia and Namibia’s Zambezi region, suggesting its localized regional circulation.^45^^,^^70^ This mutation has not been demonstrated to affect artemisinin efficacy in parasites with an African genetic background and appears to be rare across the rest of Africa. The candidate *k13* P441L marker, found in three samples from Cuando-Cubango, along the Namibia border, has been recently observed at a high prevalence in northern Namibia and in southern and central Zambia through the GenE8 project, as well as southwestern Uganda.^23^^,^^45^^,^^70^^–^^72^ Although robust clinical and laboratory evidence linking P441L to delayed clearance in Africa is lacking, a recent study in Myanmar reported a rise in its prevalence following a DHAP mass drug administration campaign.^73^

Other nonvalidated mutations previously detected in the region by the GenE8 project were also identified: the *k13* P667A mutation, recently reported at high prevalence in eastern Zambia, was found in two samples from Moxico, mirroring the geographical patterns observed for *k13* P574L. Limited laboratory data suggest that *k13* P667T/S mutations are associated with delayed clearance, pointing to a possible phenotype for *k13* P667A. The *k13* Q613E mutation, detected at low prevalence in five provinces, has been reported at low prevalence in Zambia, Angola, and the DRC.^36^^,^^74^^–^^76^ This mutation had no effect on in vitro clearance of a single laboratory isolate.^71^ Similarly, *k13* A578S, found at low prevalence in 3 provinces, has been documented across Africa without evidence of impacting artemisinin susceptibility.^77^

The clinical impact of *k13* validated and candidate markers, as well as other novel mutations, on the efficacy of ACT and artemisinin monotherapy in Angola remains to be studied. However, their low prevalence suggests that they are not an immediate concern. ACT efficacy has been monitored every two years in Angola since 2013. After molecular correction, AL efficacy dropped below 90% in four of five surveys conducted in Zaire and in the 2019 survey in Lunda Sul.^11^^–^^15^ This study did not find any *k13* markers of ART-R or mutations in the coronin gene previously linked to artemisinin resistance.^78^ These findings are consistent with the high TES day 3 clearance rates, which suggest no clinical evidence of ART-R.

Although reduced lumefantrine susceptibility has been reported in Uganda, no validated molecular marker for lumefantrine resistance exists, and there have been no reports suggesting clinically significant changes in its efficacy.^3^^,^^79^^,^^80^ The *mdr1* N86 genotype, associated with lower lumefantrine susceptibility, was found in almost all samples across all provinces and is unlikely to explain the potential reduction in AL efficacy in Zaire.^30^ Whereas AL efficacy in Zaire has remained below 90%, it has been relatively stable since 2013.^11^^–^^15^ Furthermore, molecular correction methods used in TES have inherent technical limitations that could introduce biases into these estimates.^81^^,^^82^ At the time of writing this paper, a new study reported an association between increased *mdr1* copy number and reduced AL treatment efficacy in Bengo province in northern Angola.^83^ This study did not assess *mdr1* copy number variations. Together, these data suggest that Angola may be facing emerging ACT efficacy challenges on two fronts: 1) reduced clinical efficacy of AL in the northwest and 2) the circulation of *k13* mutations in the southeast, which, though currently rare, could increase given their rising prevalence in neighboring countries.^45^^,^^70^ Angola currently recommends three ACTs (AL, ASAQ and DP) for malaria treatment, without systematic rotation or predefined geographic targeting. In line with evolving strategies in Eastern Africa and current WHO recommendations, the Angolan National Malaria Control Program is actively discussing the adoption of rotational, geography-based multiple first-line therapies to prevent and contain the spread of ACT resistance.^84^ Encouragingly, the efficacy of non-AL ACTs in the country remains very high.^11^^–^^15^

This study had several limitations. Samples were collected during a single season, restricting the assessment of temporal trends. The sampling strategy aimed to estimate country-level prevalence with broad geographic coverage, but it was not designed to detect fine-scale spatial patterns and did not cover all provinces, leaving vast geographical areas not surveyed. The exclusion of Lunda Sul province from the study reduced its geographic representativeness and prevented attainment of the overall target sample size. Delays in study initiation resulted in some health facilities in low transmission strata not achieving the required sample size. Although the majority of health facilities were randomly selected, TES sentinel sites were deliberately included, which may have violated the statistical assumptions underlying the use of survey weights to correct for sampling bias. Additionally, the low prevalence of most mutations of interest and missing data for certain demographic variables limited the sample size available for analyzing associations between mutations and demographic factors. Allele frequencies were estimated only at the municipal level, as the assumption of panmixia is unlikely to hold at larger geographic scales. Technical limitations of the molecular assay included difficulties in phasing SNPs from separate amplicons for haplotype reconstruction.

This study provides an up-to-date overview of the distribution of mutations associated with antimalarial resistance in Angola, revealing marked geographic heterogeneity: higher prevalence of SP resistance markers in the east, low levels presence of ART-R markers in the southeast, and high frequencies of markers linked to chloroquine and higher-level SP resistance in the northwest. Although the findings do not indicate an expansion of known ART-R markers that could compromise ACT efficacy, they highlight the persistent risk of selection and spread of resistance. These findings complement those from neighboring countries in the southern African region, emphasizing the importance of routine molecular surveillance and regional data sharing and coordination for timely, evidence-based policy decisions.

Although clinical evidence on the impact of the observed genotypes remains limited, the findings also underscore the need to strengthen mitigation and prevention efforts. Angola should continue to develop adaptive strategies informed by surveillance data, including coordinated cross-border elimination initiatives in areas where genotypes of concern have been detected, the rational use of multiple ACTs and consideration of triple ACTs to minimize partner drug resistance selection pressure, and advocacy for the development of alternative therapies.

## Supplemental Materials

10.4269/ajtmh.25-0226 Supplemental Materials
