# Supplementary material for: Geographical Heterogeneity in Antimalarial Resistance Markers by Genomic Surveillance in Angola, 2023
Source: Am J Trop Med Hyg. 2026 Mar 17;115(5 Suppl):19–30. doi: 10.4269/ajtmh.25-0226 (PMC13736874; doi:10.4269/ajtmh.25-0226)
Supplement: Supplementary file 1 [file tpmd250226.SD1.pdf]

Table S1: *Dhps*/*dhfr* haplotype classification.

Gene (amino acids)	Haplotype classification	Observed pure genotypes	Observed mixed genotypes under classification
dhps<br>(431,436,437,540,581,613)	Wild type	ISAKAA	
	<2 mutations	IAAKAA<br>IAGKAA<br>ISGKAA<br>VAGKAA	IAAKAA+ISAKAA<br>IAAKAA+ISGKAA<br>IAGKAA+ISAKAA<br>IAGKAA+ISGKAA<br>ISAKAA+ISGKAA<br>ISGKAA+ISGKAS<br>ISGKAA+VAGKAA<br>IAAKAA+IAGKAA+ISGKAA<br>IAGKAA+ISAKAA+ISGKAA
	Double	IAGEAA<br>ISGEAA<br>VAGEAA	IAGEAA+IAGKAA<br>IAGEAA+ISGEAA<br>ISGEAA+ISGKAA<br>ISGEAA+VAGEAA
	Triple	ISGEGA	ISGEGA+ISGKAA<br>ISGEAA+ISGEGA+ISGKAA
dhfr<br>(16,51,59,108,164)	Wild type	ANCSI	
	<3 mutations	AICNI<br>ANRNI<br>ANCNI	AICNI+ANRNI
	Triple	AIRNI	AICNI+AIRNI<br>AIRNI+ANCNI<br>AIRNI+ANRNI<br>AICNI+AIRNI+ANRNI
	Quadruple	AIRNL	
dhps-dhfr	Wild type	ISAKAA-ANCSI	
	<5 mutations	IAAKAA-AIRNI<br>IAGKAA-AICNI<br>IAGKAA-AIRNI<br>IAGKAA-ANRNI<br>ISAKAA-AICNI<br>ISAKAA-AIRNI<br>ISAKAA-ANCNI<br>ISGEAA-AICNI<br>ISGEAA-ANRNI<br>ISGKAA-AICNI<br>ISGKAA-AIRNI<br>ISGKAA-ANCSI<br>ISGKAA-ANRNI<br>VAGKAA-AIRNI	IAAKAA-AICNI+ISAKAA-AICNI<br>IAGKAA-AICNI+IAGKAA-AIRNI<br>IAGKAA-AICNI+ISGKAA-AICNI<br>IAGKAA-AIRNI+ISAKAA-AIRNI<br>IAGKAA-AIRNI+ISGKAA-AIRNI<br>ISAKAA-AIRNI+ISGKAA-AIRNI<br>ISGEAA-AICNI+ISGKAA-AICNI<br>ISGKAA-AICNI+ISGKAA-AIRNI<br>ISGKAA-AICNI+ISGKAA-ANRNI<br>ISGKAA-AICNI+ISGKAS-AICNI<br>ISGKAA-AIRNI+ISGKAA-ANRNI<br>ISGKAA-AIRNI+VAGKAA-AIRNI<br>ISGKAA-AICNI+ISGKAA-AIRNI+ISGKAA-ANRNI
	Quintuple	IAGEAA-AIRNI<br>ISGEAA-AIRNI	IAGEAA-AIRNI+IAGKAA-AIRNI<br>IAGEAA-AIRNI+ISGEAA-AIRNI<br>ISGEAA-AICNI+ISGEAA-AIRNI<br>ISGEAA-AIRNI+ISGEAA-ANRNI<br>ISGEAA-AIRNI+ISGKAA-AIRNI<br>ISGEAA-AIRNI+VAGEAA-AIRNI<br>ISGEAA-AICNI+ISGEAA-AIRNI+ISGEAA-ANRNI
	Sextuple	ISGEGA-AIRNI	ISGEGA-AIRNI+ISGKAA-AIRNI<br>ISGEAA-AIRNI+ISGEGA-AIRNI+ISGKAA-AIRNI

Table S2. Proportion of *P. falciparum* infections and coinfections with non-*falciparum* species

	**Overall (N = 820)**	**Zaire (N = 134)**	**Uíge (N = 124)**	**Bié (N = 136)**	**Moxico (N = 116)**	**Benguela (N = 128)**	**Namibe (N = 68)**	**Cuando Cubango (N = 114)**
**Pf**	774 (94.39%)	128 (95.52%)	114 (91.94%)	128 (94.12%)	105 (90.52%)	124 (96.88%)	66 (97.06%)	109 (95.61%)
**Pf+Pm**	28 (3.41%)	4 (2.99%)	8 (6.45%)	4 (2.94%)	6 (5.17%)	3 (2.34%)	2 (2.94%)	1 (0.88%)
**Pf+Pm+ Poc+Pow**	1 (0.12%)	0 (0%)	0 (0%)	1 (0.74%)	0 (0%)	0 (0%)	0 (0%)	0 (0%)
**Pf+Pm+ Pow**	2 (0.24%)	0 (0%)	0 (0%)	0 (0%)	0 (0%)	0 (0%)	0 (0%)	2 (1.75%)
**Pf+Poc**	7 (0.85%)	1 (0.75%)	0 (0%)	0 (0%)	4 (3.45%)	1 (0.78%)	0 (0%)	1 (0.88%)
**Pf+Poc+ Pow**	2 (0.24%)	0 (0%)	1 (0.81%)	1 (0.74%)	0 (0%)	0 (0%)	0 (0%)	0 (0%)
**Pf+Pow**	6 (0.73%)	1 (0.75%)	1 (0.81%)	2 (1.47%)	1 (0.86%)	0 (0%)	0 (0%)	1 (0.88%)

Pf: *P. falciparum*. Pm: *P. malariae*. Poc: *P. ovale curtisi*. Pow: *P. ovale wallikeri*. Pv: *P. vivax*.

Table S3. Demographic characteristics of participants whose samples were qPCR-positive and successfully sequenced*.

Characteristic	qPCR-positive	Successfully sequenced	p-value**
**N**	1254	722	
**Age**			**<0.001**
< 5 years	236 (19%)	168 (23%)	
5 - 14 years	428 (34%)	287 (40%)	
15 - 24 years	293 (23%)	156 (22%)	
≥25 years	297 (24%)	111 (15%)	
**Gender**			0.221
Female	704 (56%)	384 (53%)	
Male	550 (44%)	338 (47%)	
**Occupation**			**<0.001**
Agricultural	193 (15%)	66 (9.2%)	
Minor	371 (30%)	257 (36%)	
Other	151 (12%)	68 (9.5%)	
Student	467 (37%)	297 (41%)	
Unemployed	64 (5.1%)	30 (4.2%)	
Unknown	8	4	
**Travel**			0.465
Domestic	74 (5.9%)	49 (6.8%)	
International	1 (<0.1%)	1 (0.1%)	
No Travel	1,178 (94%)	672 (93%)	
Unknown	1	0	
**Province**			0.526
Benguela	160 (13%)	113 (16%)	
Bié	216 (17%)	118 (16%)	
Cuando Cubango	162 (13%)	93 (13%)	
Moxico	200 (16%)	105 (15%)	
Namibe	102 (8.1%)	57 (7.9%)	
Uíge	205 (16%)	105 (15%)	
Zaire	209 (17%)	131 (18%)	

*:with a valid genotype in all 53 assessed *k13* SNPs.
**chi-squared test

Table S4: Adjusted national prevalence for mutations observed in >0.5% of the samples. Source data for Figures 2A and 3B.

Gene	SNP	Prevalence (95% CI)	N	Design effect	Intraclass correlation coefficient
crt	72-76 CVIET	17.71% (3.49-44.8%)	820	39.2	0.66
crt	A220S	10.03% (1.94-27.48%)	226	6.15	0.34
crt	I356T	12.21% (2.02-34.53%)	770	28.94	0.52
dhps	I431V	0.44% (0.01-2.53%)	804	2.82	0.03
dhps	S436A	33.31% (23.07-44.84%)	804	7.67	0.12
dhps	A437G	98.09% (94.35-99.63%)	804	4.08	0.05
dhps	K540E	43.5% (33.99-53.37%)	761	5.27	0.08
dhps	A581S	3.58% (0.94-9.12%)	808	5.73	0.08
dhps	A613S	0.64% (0.07-2.41%)	805	2.1	0.02
dhfr	N51I	99.56% (98.57-99.93%)	768	1.06	0
dhfr	C59R	87.31% (78.96-93.22%)	766	5.98	0.09
dhfr	S108N	99.94% (99.53-100%)	802	0.59	-0.01
k13	P441L	0.09% (0-0.69%)	768	0.81	0
k13	A578S	0.71% (0.1-2.35%)	810	1.87	0.02
k13	Q613E	1.57% (0.23-5.17%)	810	4.17	0.06
k13	P667A	0.18% (0-1.45%)	732	1.64	0.01
mdr1	N86	98.09% (96.91-98.91%)	649	0.59	-0.01
mdr1	N86Y	0.72% (0.17-1.94%)	649	1	0
mdr1	Y184F	53.2% (48.3-58.06%)	788	1.41	0.01
mdr2	I492V	61.11% (53.64-68.23%)	739	3.04	0.04
PF3D7_1322700	T236I	1.34% (0.32-3.6%)	658	1.89	0.02

Table S5: Unadjusted proportion of samples carrying a mutation at the provincial level for mutations observed in >0.5% of the samples.
% (number of samples carrying the mutation/total number of samples with a valid genotype)

Gene	SNP	Benguela	Bie	Cuando Cubango	Moxico	Namibe	Uige	Zaire
crt	72-76 CVIET	3.12% (4/128)	5.88% (8/136)	1.75% (2/114)	0.86% (1/116)	4.41% (3/68)	58.87% (73/124)	48.51% (65/134)
crt	A220S	2.63% (1/38)	2.17% (1/46)	0% (0/10)	0% (0/37)	7.14% (1/14)	27.59% (8/29)	32.69% (17/52)
crt	I356T	1.67% (2/120)	0.8% (1/125)	0% (0/102)	0.92% (1/109)	3.03% (2/66)	44.44% (52/117)	35.11% (46/131)
dhps	I431V	0% (0/122)	0% (0/131)	0% (0/113)	0% (0/116)	0% (0/67)	0% (0/121)	2.99% (4/134)
dhps	S436A	18.85% (23/122)	49.62% (65/131)	43.36% (49/113)	25% (29/116)	16.42% (11/67)	44.63% (54/121)	35.07% (47/134)
dhps	A437G	99.18% (121/122)	100% (131/131)	99.12% (112/113)	95.69% (111/116)	100% (67/67)	95.87% (116/121)	98.51% (132/134)
dhps	K540E	40.87% (47/115)	55.65% (69/124)	58.82% (60/102)	43.24% (48/111)	40.62% (26/64)	27.68% (31/112)	37.59% (50/133)
dhps	A581G	0.8% (1/125)	1.53% (2/131)	0.88% (1/113)	2.59% (3/116)	0% (0/68)	4.13% (5/121)	14.18% (19/134)
dhps	A613S	0% (0/125)	0% (0/131)	0% (0/111)	0% (0/116)	1.47% (1/68)	0% (0/120)	2.99% (4/134)
dhfr	N51I	100% (117/117)	99.19% (123/124)	100% (105/105)	98.21% (110/112)	100% (64/64)	100% (113/113)	99.25% (132/133)
dhfr	C59R	78.45% (91/116)	87.9% (109/124)	88.57% (93/105)	91.96% (103/112)	73.44% (47/64)	96.43% (108/112)	90.98% (121/133)
dhfr	S108N	100% (122/122)	100% (130/130)	100% (113/113)	100% (116/116)	100% (66/66)	100% (121/121)	99.25% (133/134)
k13	P441L	0% (0/115)	0% (0/125)	2.8% (3/107)	0% (0/110)	0% (0/64)	0% (0/114)	0% (0/133)
k13	A578S	0.8% (1/125)	2.27% (3/132)	0% (0/113)	1.72% (2/116)	0% (0/68)	0% (0/122)	0% (0/134)
k13	Q613E	2.4% (3/125)	0.76% (1/132)	4.42% (5/113)	0% (0/116)	1.47% (1/68)	0% (0/122)	0% (0/134)
k13	P667A	0% (0/114)	0% (0/120)	0% (0/95)	1.9% (2/105)	0% (0/60)	0% (0/106)	0% (0/132)
mdr1	Y184F	49.17% (59/120)	54.26% (70/129)	46.85% (52/111)	50.86% (59/116)	59.68% (37/62)	56.41% (66/117)	48.87% (65/133)
mdr1	N86	100% (105/105)	99.07% (106/107)	100% (73/73)	100% (91/91)	98.04% (50/51)	98.94% (93/94)	98.44% (126/128)
mdr1	N86Y	0.95% (1/105)	1.87% (2/107)	1.37% (1/73)	1.1% (1/91)	1.96% (1/51)	1.06% (1/94)	4.69% (6/128)
mdr2	I492V	64.66% (75/116)	63.33% (76/120)	54.17% (52/96)	69.16% (74/107)	45% (27/60)	59.63% (65/109)	67.94% (89/131)
PF3D7_1322700	T236I	4.85% (5/103)	0.95% (1/105)	0% (0/90)	1.08% (1/93)	2% (1/50)	1.02% (1/98)	0% (0/119)

Table S6: Unadjusted proportion of samples carrying a mutation at the health facility level for mutations observed in >0.5% of the samples.
% (number of samples carrying the mutation/total number of samples with a valid genotype)

Gene	SNP	Centro de Saude Txizainga (Saurimo, Lunda Sul)	Centro de Saude do Quitexe (Quitexe, Uige)	Centro de Saude 5 de Abril (Namibe, Namibe)	Centro de Saude Piloto (Kuito, Bie)	Centro de Saude da Zona Sul (Tombwa, Namibe)	Centro de Saude Kianganga (M'Banza Congo, Zaire)	Hospital Geral de Benguela (Benguela, Benguela)	Hospital Geral de Menongue (Menongue, Cuando Cubango)	Hospital Municipal da Damba (Damba, Uige)	Hospital Municipal de Benguela (Benguela, Benguela)	Hospital Municipal de Mbanza Congo (M'Banza Congo, Zaire)	Hospital Municipal de Nhareia (Nharea, Bie)	Hospital Municipal do Calai (Calai, Cuando Cubango)	Hospital Municipal do Luau (Luau, Moxico)	Hospital Municipal do Luena (Moxico, Moxico)
crt	72-76 CVIET	5.88% (4/68)	52.24% (35/67)	4.69% (3/64)	8.33% (6/72)	0% (0/4)	47.89% (34/71)	4.35% (3/69)	1.41% (1/71)	66.67% (38/57)	1.69% (1/59)	49.21% (31/63)	3.12% (2/64)	2.33% (1/43)	0% (0/57)	1.69% (1/59)
crt	A220S	10.34% (3/29)	20% (2/10)	7.69% (1/13)	0% (0/19)	0% (0/1)	16.67% (3/18)	3.23% (1/31)	0% (0/4)	31.58% (6/19)	0% (0/7)	41.18% (14/34)	3.7% (1/27)	0% (0/6)	0% (0/25)	0% (0/12)
crt	I356T	0% (0/65)	33.87% (21/62)	3.23% (2/62)	1.47% (1/68)	0% (0/4)	37.14% (26/70)	1.52% (1/66)	0% (0/62)	56.36% (31/55)	1.85% (1/54)	32.79% (20/61)	0% (0/57)	0% (0/40)	0% (0/56)	1.89% (1/53)
dhps	I431V	0% (0/63)	0% (0/66)	0% (0/63)	0% (0/72)	0% (0/4)	2.82% (2/71)	0% (0/64)	0% (0/71)	0% (0/55)	0% (0/58)	3.17% (2/63)	0% (0/59)	0% (0/42)	0% (0/57)	0% (0/59)
dhps	S436A	14.29% (9/63)	54.55% (36/66)	17.46% (11/63)	50% (36/72)	0% (0/4)	36.62% (26/71)	17.19% (11/64)	45.07% (32/71)	32.73% (18/55)	20.69% (12/58)	33.33% (21/63)	49.15% (29/59)	40.48% (17/42)	28.07% (16/57)	22.03% (13/59)
dhps	A437G	96.83% (61/63)	98.48% (65/66)	100% (63/63)	100% (72/72)	100% (4/4)	97.18% (69/71)	100% (64/64)	98.59% (70/71)	92.73% (51/55)	98.28% (57/58)	100% (63/63)	100% (59/59)	100% (42/42)	91.23% (52/57)	100% (59/59)
dhps	K540E	46.77% (29/62)	36.84% (21/57)	41.67% (25/60)	67.65% (46/68)	25% (1/4)	38.03% (27/71)	46.03% (29/63)	59.38% (38/64)	18.18% (10/55)	34.62% (18/52)	37.1% (23/62)	41.07% (23/56)	57.89% (22/38)	42.86% (24/56)	43.64% (24/55)
dhps	A581G	0% (0/66)	3.03% (2/66)	0% (0/64)	2.78% (2/72)	0% (0/4)	15.49% (11/71)	0% (0/66)	1.41% (1/71)	5.45% (3/55)	1.69% (1/59)	12.7% (8/63)	0% (0/59)	0% (0/42)	5.26% (3/57)	0% (0/59)
dhps	A613S	0% (0/64)	0% (0/65)	1.56% (1/64)	0% (0/72)	0% (0/4)	2.82% (2/71)	0% (0/66)	0% (0/70)	0% (0/55)	0% (0/59)	3.17% (2/63)	0% (0/59)	0% (0/41)	0% (0/57)	0% (0/59)
dhfr	N51I	98.39% (61/62)	100% (59/59)	100% (60/60)	98.53% (67/68)	100% (4/4)	98.59% (70/71)	100% (62/62)	100% (65/65)	100% (54/54)	100% (55/55)	100% (62/62)	100% (56/56)	100% (40/40)	98.21% (55/56)	98.21% (55/56)
dhfr	C59R	82.26% (51/62)	98.28% (57/58)	75% (45/60)	92.65% (63/68)	50% (2/4)	90.14% (64/71)	74.19% (46/62)	92.31% (60/65)	94.44% (51/54)	83.33% (45/54)	91.94% (57/62)	82.14% (46/56)	82.5% (33/40)	92.86% (52/56)	91.07% (51/56)
dhfr	S108N	98.44% (63/64)	100% (66/66)	100% (62/62)	100% (71/71)	100% (4/4)	98.59% (70/71)	100% (64/64)	100% (71/71)	100% (55/55)	100% (58/58)	100% (63/63)	100% (59/59)	100% (42/42)	100% (57/57)	100% (59/59)
k13	P441L	0% (0/62)	0% (0/59)	0% (0/60)	0% (0/69)	0% (0/4)	0% (0/71)	0% (0/63)	0% (0/66)	0% (0/55)	0% (0/52)	0% (0/62)	0% (0/56)	7.32% (3/41)	0% (0/56)	0% (0/54)
k13	A578S	7.58% (5/66)	0% (0/67)	0% (0/64)	4.17% (3/72)	0% (0/4)	0% (0/71)	1.52% (1/66)	0% (0/71)	0% (0/55)	0% (0/59)	0% (0/63)	0% (0/60)	0% (0/42)	1.75% (1/57)	1.69% (1/59)
k13	Q613E	1.52% (1/66)	0% (0/67)	1.56% (1/64)	0% (0/72)	0% (0/4)	0% (0/71)	1.52% (1/66)	7.04% (5/71)	0% (0/55)	3.39% (2/59)	0% (0/63)	1.67% (1/60)	0% (0/42)	0% (0/57)	0% (0/59)
k13	P667A	0% (0/62)	0% (0/54)	0% (0/56)	0% (0/66)	0% (0/4)	0% (0/70)	0% (0/63)	0% (0/60)	0% (0/52)	0% (0/51)	0% (0/62)	0% (0/54)	0% (0/35)	0% (0/56)	4.08% (2/49)
mdr1	Y184F	47.62% (30/63)	66.13% (41/62)	58.62% (34/58)	55.71% (39/70)	75% (3/4)	50.7% (36/71)	45.31% (29/64)	50.72% (35/69)	45.45% (25/55)	53.57% (30/56)	46.77% (29/62)	52.54% (31/59)	40.48% (17/42)	63.16% (36/57)	38.98% (23/59)
mdr1	N86	100% (59/59)	97.62% (41/42)	97.87% (46/47)	98.18% (54/55)	100% (4/4)	97.1% (67/69)	100% (61/61)	100% (48/48)	100% (52/52)	100% (44/44)	100% (59/59)	100% (52/52)	100% (25/25)	100% (55/55)	100% (36/36)
mdr1	N86Y	0% (0/59)	2.38% (1/42)	2.13% (1/47)	3.64% (2/55)	0% (0/4)	5.8% (4/69)	1.64% (1/61)	2.08% (1/48)	0% (0/52)	0% (0/44)	3.39% (2/59)	0% (0/52)	0% (0/25)	1.82% (1/55)	0% (0/36)
mdr2	I492V	66.67% (40/60)	62.5% (35/56)	48.21% (27/56)	67.19% (43/64)	0% (0/4)	67.14% (47/70)	65.62% (42/64)	56.67% (34/60)	56.6% (30/53)	63.46% (33/52)	68.85% (42/61)	58.93% (33/56)	50% (18/36)	75% (42/56)	62.75% (32/51)
PF3D7_ 1322700	T236I	0% (0/57)	0% (0/49)	2.17% (1/46)	0% (0/57)	0% (0/4)	0% (0/62)	6.78% (4/59)	0% (0/53)	2.04% (1/49)	2.27% (1/44)	0% (0/57)	2.08% (1/48)	0% (0/37)	0% (0/51)	2.38% (1/42)

Table S7: Municipal-level mutation allele frequencies. Source data for Figure 2B and 3C.

Gene	Codon(s)	Allele	Benguela (Benguela)	Calai (Cuando Cubango)	Damba (Uige)	Kuito (Bie)	Luau (Moxico)	MBanza Congo (Zaire)	Menongue (Cuando Cubango)	Moxico (Moxico)	Namibe (Namibe)	Nharea (Bie)	Quitexe (Uige)
crt	72-76	CVIET	1.13% (0.23-2.91%)	0%	52.36% (41.6-63.52%)	2.05% (0.75-4.62%)	0%	34% (27.35-41.17%)	0%	0%	1.63% (0.21-5.2%)	0.09% (0-2.31%)	21.5% (15.05-29.13%)
crt	72-76	CVMNK	98.87% (97.09-99.77%)	100%	47.64% (36.48-58.4%)	97.95% (95.38-99.25%)	100%	66% (58.83-72.65%)	100%	100%	98.37% (94.8-99.79%)	99.91% (97.69-100%)	78.5% (70.87-84.95%)
crt	220	A	100%	100%	74.45% (56.34-87.62%)	100%	100%	79.44% (69.5-86.96%)	100%	100%	100%	100%	97.76% (88.87-99.93%)
crt	220	S	0%	0%	25.55% (12.38-43.66%)	0%	0%	20.56% (13.04-30.5%)	0%	0%	0%	0%	2.24% (0.07-11.13%)
crt	356	I	99.71% (98.59-99.99%)	100%	57.32% (44.98-68.25%)	100%	100%	77.36% (70.74-82.93%)	100%	100%	99.3% (96.33-100%)	100%	88.13% (82.65-92.42%)
crt	356	T	0.29% (0.01-1.41%)	0%	42.68% (31.75-55.02%)	0%	0%	22.64% (17.07-29.26%)	0%	0%	0.7% (0-3.67%)	0%	11.87% (7.58-17.35%)
dhps	431/436/437	IAG	9.66% (6.12-13.95%)	20.56% (16.82-34.88%)	21.65% (13.95-31.14%)	21.63% (15.68-29.46%)	9.35% (4.88-15.46%)	19.6% (14.72-25.42%)	21.67% (15.17-29.22%)	12.6% (6.88-20.37%)	9.7% (5.14-16.95%)	26.51% (18.91-35.03%)	23.07% (15.98-31.27%)
dhps	431/436/437	ISA	0% (0-0.06%)	0% (0-0.26%)	6.94% (2.59-13.69%)	0.69% (0.08-2.51%)	10.5% (5.99-17.06%)	1.99% (0.63-4.2%)	0.99% (0.11-3.04%)	0.81% (0.04-3.88%)	0.69% (0.02-3.9%)	1.14% (0.16-3.95%)	3.76% (1.81-7.27%)
dhps	431/436/437	ISG	90.34% (86.01-93.88%)	79.44% (65.12-83.11%)	71.25% (60.6-79.86%)	77.47% (69.47-83.59%)	76.81% (68.71-84.27%)	77.12% (70.97-82.25%)	77.27% (69.58-83.73%)	86.33% (78.61-92.26%)	89.25% (81.8-94.03%)	72.21% (63.74-79.39%)	72.59% (63.74-79.7%)
dhps	431/436/437	VAG	0%	0%	0%	0%	0%	1.12% (0.25-2.95%)	0% (0-0.5%)	0%	0%	0%	0%
dhps	431/436/437	IAA	0%	0% (0-0.01%)	0%	0% (0-0.29%)	2.71% (0.72-6.42%)	0%	0%	0%	0%	0%	0.31% (0.02-1.7%)
dhps	540/581	EA	25.16% (19.29-32.18%)	37.91% (27-52.03%)	9.35% (4.35-16.72%)	42.23% (32.99-51.32%)	25.36% (16.76-35.36%)	17.97% (12.92-23.56%)	39.21% (30.13-49.44%)	35.13% (24.8-46.59%)	28.62% (19.35-38.72%)	23.52% (16.31-32.23%)	12.44% (10-17.08%)
dhps	540/581	KA	74.84% (67.82-80.71%)	62.09% (47.97-73%)	89.61% (81.97-94.98%)	57.32% (47.96-66.53%)	73.19% (63.3-82.4%)	74.66% (68.65-80.11%)	60.79% (50.56-69.87%)	64.87% (53.41-75.2%)	71.38% (61.28-80.65%)	76.48% (67.77-83.69%)	87.56% (82.92-90%)
dhps	540/581	EG	0%	0%	0.77% (0.03-3.83%)	0.3% (0.01-1.61%)	1.22% (0.2-3.83%)	5.97% (3.22-9.62%)	0%	0%	0%	0%	0%
dhps	540/581	KG	0%	0%	0%	0%	0%	1.11% (0.26-3.1%)	0%	0%	0%	0%	0%
dhps	613	A	100%	100%	100%	100%	100%	98.92% (97.02-99.76%)	100%	100%	100%	100%	100%
dhps	613	S	0%	0%	0%	0%	0%	1.08% (0.24-2.98%)	0%	0%	0%	0%	0%
dhfr	16/51/59	AIC	29.8% (23.8-36.6%)	25.77% (16.68-37.13%)	15.37% (7.89-24.33%)	25.96% (18.81-33.74%)	12.22% (7.01-18.91%)	8.98% (5.73-13.22%)	23.47% (16.8-32.15%)	14.59% (8.28-22.82%)	28.9% (20.59-38.07%)	33.9% (25.03-44.66%)	21.22% (14.87-28.93%)
dhfr	16/51/59	AIR	68.66% (61.75-74.72%)	72.98% (61.31-82.4%)	84.63% (75.67-92.11%)	70.38% (62.38-77.41%)	82.92% (75.66-89.07%)	90.96% (86.75-94.14%)	75.33% (66.8-82.15%)	82.04% (73.91-89.04%)	70.13% (60.84-78.68%)	63.83% (52.98-73.24%)	76.88% (68.71-83.46%)

dhfr	16/51/59	ANR	1.45% (0.48-3.49%)	0.89% (0.03-4.22%)	0%	3.63% (1.63-6.57%)	3.84% (1.29-8.08%)	0% (0-0.34%)	0.9% (0.15-3.09%)	2.97% (0.8-7.7%)	0.71% (0.02-3.66%)	1.92% (0.42-5.26%)	1.79% (0.57-4.1%)
dhfr	16/51/59	ANC	0%	0%	0%	0%	0.51% (0.02-2.69%)	0% (0-0.28%)	0%	0%	0%	0%	0%
dhfr	108/164	NI	100%	100%	100%	100%	100%	100%	100%	100%	100%	100%	100%
dhfr	108/164	SI	0%	0%	0%	0%	0%	0%	0%	0%	0%	0%	0%
k13	441	L	0%	2.12% (0.28-6.68%)	0%	0%	0%	0%	0%	0%	0%	0%	0%
k13	441	P	100%	97.88% (93.32-99.72%)	100%	100%	100%	100%	100%	100%	100%	100%	100%
k13	578	A	100%	100%	100%	99.24% (97.4-99.89%)	100%	100%	100%	100%	100%	100%	100%
k13	578	S	0%	0%	0%	0.76% (0.11-2.6%)	0%	0%	0%	0%	0%	0%	0%
k13	613	E	0.69% (0.1-2.21%)	0%	0%	0%	0%	0%	2.12% (0.61-4.78%)	0%	0%	0%	0%
k13	613	Q	99.31% (97.79-99.9%)	100%	100%	100%	100%	100%	97.88% (95.22-99.39%)	100%	100%	100%	100%
k13	667	A	0%	0%	0%	0%	0%	0%	0%	0.8% (0.03-4.27%)	0%	0%	0%
k13	667	P	100%	100%	100%	100%	100%	100%	100%	99.2% (95.73-99.97%)	100%	100%	100%
mdr1	86	N	100%	100%	100%	99.62% (98-99.98%)	100%	97.9% (95.51-99.34%)	100%	100%	100%	100%	100%
mdr1	86	Y	0%	0%	0%	0.38% (0.02-2%)	0%	2.1% (0.66-4.49%)	0%	0%	0%	0%	0%
mdr1	184	F	33.37% (26.56-41.24%)	25.86% (15.21-37.51%)	36.42% (25.18-48.33%)	26.74% (19.87-35.63%)	40.79% (30.16-51.54%)	34.01% (27.58-41.37%)	30.8% (22.65-40.17%)	28.36% (18.94-39.54%)	45.5% (34.59-56.73%)	29.63% (21.41-38.42%)	33.99% (24.81-43.23%)
mdr1	184	Y	66.63% (58.76-73.44%)	74.14% (62.49-84.79%)	63.58% (51.67-74.82%)	73.26% (64.37-80.13%)	59.21% (48.46-69.84%)	65.99% (58.63-72.42%)	69.2% (59.83-77.35%)	71.64% (60.46-81.06%)	54.5% (43.27-65.41%)	70.37% (61.58-78.59%)	66.01% (56.77-75.19%)
mdr2	492	I	53.23% (45.34-60.88%)	63.86% (50.39-76.58%)	52.48% (40.27-63.47%)	69.16% (60.58-77.1%)	44.81% (33.66-55.91%)	40.77% (34.04-47.87%)	65.59% (55.84-74.43%)	48.17% (35.4-59.87%)	67.16% (56.29-76.8%)	59.24% (47.87-69.86%)	64.3% (53.09-74.1%)
mdr2	492	V	46.77% (39.12-54.66%)	36.14% (23.42-49.61%)	47.52% (36.53-59.73%)	30.84% (22.9-39.42%)	55.19% (44.09-66.34%)	59.23% (52.13-65.96%)	34.41% (25.57-44.16%)	51.83% (40.13-64.6%)	32.84% (23.2-43.71%)	40.76% (30.14-52.13%)	35.7% (25.9-46.91%)
PF3D7_1322700	236	I	1.72% (0.54-3.96%)	0%	0%	0%	0%	0%	0%	0%	0%	0%	0%
PF3D7_1322700	236	T	98.28% (96.04-99.46%)	100%	100%	100%	100%	100%	100%	100%	100%	100%	100%

Table S8: Proportion of samples carrying a *crt* haplotype.

Number of samples carrying the haplotype (% of total, N)

	Country	Benguela	Bie	Cuando Cubango	Moxico	Namibe	Uige	Zaire
crt **72-76/220/356 haplotype**	N=226	38	46	10	37	14	29	52
CVIETAI+CVMNKAI	7 (3.1%)	1 (2.63%)	0	1 (10%)	0	0	4 (13.79%)	1 (1.92%)
CVIETSI	3 (1.33%)	0	0	0	0	0	0	3 (5.77%)
CVIETSI+CVIETST	2 (0.88%)	0	0	0	0	0	1 (3.45%)	1 (1.92%)
CVIETST	13 (5.75%)	0	0	0	0	0	6 (20.69%)	7 (13.46%)
CVMNKAI	182 (80.53%)	35 (92.11%)	45 (97.83%)	9 (90%)	37 (100%)	12 (85.71%)	15 (51.72%)	29 (55.77%)
CVMNKAI+CVMNKSI	1 (0.44%)	1 (2.63%)	0	0	0	0	0	0
CVMNKAI+CVIETAI	1 (0.44%)	0	0	0	0	1 (7.14%)	0	0
Undetermined[a]	17 (7.52%)	1 (2.63%)	1 (2.17%)	0	0	1 (7.14%)	3 (10.34%)	11 (21.15%)

[a]Mixed genotype in more than 2 targets.

Table S9: Mixed-effects univariate logistic regression of select demographic characteristics and genotypes, and random effects for health facilities

		**Age**					**Gender**			**Occupation**					
		**N**	< 5 years	5 - 14 years	15 - 24 years	≥25 years	**N**	Female	Male	**N**	Minor	Student	Agricultural	Other	Unemployed
***crt* 72-76 CVIET**	n/N (%)		26/181 (14.36%)	52/322 (16.15%)	40/175 (22.86%)	38/142 (26.76%)		80/434 (18.43%)	76/386 (19.69%)		45/277 (16.25%)	59/332 (17.77%)	32/85 (37.65%)	13/84 (15.48%)	5/37 (13.51%)
	OR (95% CI)	820	Ref.	1.14 (0.62-2.11)	1.30 (0.67-2.53)	**2.52 (1.25-5.07)**	820	Ref.	1.16 (0.75-1.78)	815	Ref.	0.85 (0.50-1.44)	**2.34 (1.13-4.85)**	1.71 (0.72-4.04)	2.53 (0.74-8.06)
	p-value			0.7	0.4	**0.01**			0.6			0.6	**0.022**	0.2	0.14
***crt* A220S**	n/N (%)		6/62 (9.68%)	13/103 (12.62%)	5/37 (13.51%)	4/24 (16.67%)		14/129 (10.85%)	14/97 (14.43%)		10/96 (10.42%)	13/96 (13.54%)	2/15 (13.33%)	0/12 (0%)	2/6 (33.33%)
	OR (95% CI)	226	Ref.	1.10 (0.35-3.59)	0.70 (0.17-2.94)	1.36 (0.29-6.52)	226	Ref.	1.09 (0.44-2.70)	225	NA	NA	NA	NA	NA
	p-value			0.9	0.6	0.7			0.8		NA	NA	NA	NA	NA
***crt* I356T**	n/N (%)		17/176 (9.66%)	38/299 (12.71%)	25/165 (15.15%)	24/130 (18.46%)		55/409 (13.45%)	49/361 (13.57%)		30/267 (11.24%)	42/309 (13.59%)	20/76 (26.32%)	7/79 (8.86%)	3/34 (8.82%)
	OR (95% CI)	770	Ref.	1.38 (0.68-2.82)	1.15 (0.53-2.46)	**2.29 (1.02-5.16)**	770	Ref.	1.04 (0.64-1.72)	765	Ref.	0.96 (0.53-1.75)	2.27 (0.99-5.22)	1.11 (0.38-3.19)	2.66 (0.55-12.9)
	p-value			0.3	0.13	**0.044**			0.9			0.9	0.052	0.9	0.2
***dhps* S436A**	n/N (%)		64/180 (35.56%)	101/311 (32.48%)	65/173 (37.57%)	48/140 (34.29%)		135/429 (31.47%)	143/375 (38.13%)		96/273 (35.16%)	105/323 (32.51%)	37/82 (45.12%)	25/84 (29.76%)	13/37 (35.14%)
	OR (95% CI)	804	Ref.	0.96 (0.64-1.44)	1.14 (0.72-1.80)	0.86 (0.53-1.40)	804	Ref.	1.35 (1.00-1.83)	799	Ref.	1.00 (0.69-1.44)	1.15 (0.68-1.97)	0.85 (0.49-1.48)	1.02 (0.48-2.15)
	p-value			0.8	0.6	0.5			0.05			>0.9	0.6	0.6	>0.9
***dhps* A437G**	n/N (%)		177/180 (98.33%)	304/311 (97.75%)	170/173 (98.27%)	139/140 (99.29%)		424/429 (98.83%)	366/375 (97.6%)		268/273 (98.17%)	316/323 (97.83%)	82/82 (100%)	82/84 (97.62%)	37/37 (100%)
	OR (95% CI)	804	Ref.	0.50 (0.12-2.17)	0.81 (0.15-4.37)	2.29 (0.23-22.8)	804	Ref.	0.43 (0.14-1.33)	799	NA	NA	NA	NA	NA
	p-value			0.4	0.8	0.5			0.14		NA	NA	NA	NA	NA
***dhps* K540E**	n/N (%)		69/172 (40.12%)	128/296 (43.24%)	65/166 (39.16%)	69/127 (54.33%)		166/406 (40.89%)	165/355 (46.48%)		109/263 (41.44%)	131/310 (42.26%)	39/73 (53.42%)	38/76 (50%)	12/34 (35.29%)
	OR (95% CI)	761	Ref.	1.15 (0.77-1.71)	0.98 (0.62-1.55)	**1.76 (1.09-2.83)**	761	Ref.	1.26 (0.94-1.69)	756	Ref.	1.07 (0.75-1.52)	**1.81 (1.04-3.17)**	1.33 (0.78-2.28)	0.63 (0.29-1.36)
	p-value			0.5	>0.9	**0.021**			0.13			0.7	**0.037**	0.3	0.2

***dhps* A581G**	n/N (%)	808	6/179 (3.35%)	9/315 (2.86%)	8/175 (4.57%)	8/139 (5.76%)	808	15/430 (3.49%)	16/378 (4.23%)	803	9/273 (3.3%)	13/327 (3.98%)	6/82 (7.32%)	2/84 (2.38%)	1/37 (2.7%)
	OR (95% CI)		Ref.	0.86 (0.29-2.55)	1.36 (0.44-4.16)	1.74 (0.57-5.34)		Ref.	1.13 (0.53-2.37)		Ref.	0.95 (0.38-2.38)	2.77 (0.88-8.75)	0.76 (0.15-3.76)	1.10 (0.13-9.55)
	p-value			0.8	0.6	0.3			0.8			>0.9	0.082	0.7	>0.9
***mdr1* Y184F**	n/N (%)	788	91/178 (51.12%)	145/304 (47.7%)	95/170 (55.88%)	77/136 (56.62%)	788	228/423 (53.9%)	180/365 (49.32%)	783	134/270 (49.63%)	156/317 (49.21%)	46/79 (58.23%)	48/80 (60%)	19/37 (51.35%)
	OR (95% CI)		Ref.	0.88 (0.61-1.28)	1.22 (0.80-1.87)	1.24 (0.80-1.86)		Ref.	0.83 (0.62-1.10)		Ref.	0.99 (0.71-1.38)	1.40 (0.84-2.34)	1.52 (0.92-2.53)	1.08 (0.54-2.15)
	p-value			0.5	0.4	0.4			0.2			>0.9	0.2	0.11	0.8
***mdr2* I492V**	n/N (%)	739	115/170 (67.65%)	168/292 (57.53%)	90/157 (57.32%)	85/120 (70.83%)	739	251/400 (62.75%)	207/339 (61.06%)	734	168/260 (64.62%)	173/301 (57.48%)	48/72 (66.67%)	47/68 (69.12%)	18/33 (54.55%)
	OR (95% CI)		Ref.	**0.65 (0.44-0.96)**	0.64 (0.41-1.01)	1.16 (0.70-1.93)		Ref.	0.93 (0.69-1.26)		Ref.	0.74 (0.53-1.05)	1.11 (0.64-1.95)	1.24 (0.70-2.22)	0.66 (0.32-1.39)
	p-value			**0.032**	0.058	0.6			0.6			0.091	0.7	0.5	0.3
**any *k13* non-synonymous mutation**	n/N (%)	810	4/180 (2.22%)	8/315 (2.54%)	6/175 (3.43%)	4/140 (2.86%)	810	12/431 (2.78%)	10/379 (2.64%)	805	6/274 (2.19%)	9/327 (2.75%)	5/83 (6.02%)	2/84 (2.38%)	0/37 (0%)
	OR (95% CI)		Ref.	1.12 (0.33-3.82)	1.55 (0.42-5.72)	1.32 (0.32-5.44)		Ref.	0.94 (0.40-2.22)		NA	NA	NA	NA	NA
	p-value			0.9	0.5	0.7			0.9		NA	NA	NA	NA	NA

Table S10: Mixed-effects logistic regression of genotype outcomes with variables age and gender, and random effects for health facilities

			**Age**				**Gender**	
		**N**	< 5 years	5 - 14 years	15 - 24 years	≥25 years	Female	Male
**crt 72-76 CVIET**	n/N (%)	820	26/181 (14.36%)	52/322 (16.15%)	40/175 (22.86%)	38/142 (26.76%)	80/434 (18.43%)	76/386 (19.69%)
	OR (95% CI)		Ref.	1.15 (0.62-2.14)	1.36 (0.69-2.65)	**2.66 (1.31-5.41)**	Ref.	1.29 (0.83-2.00)
	p-value			0.7	0.4	**0.007**		0.3
**crt A220S**	n/N (%)	226	6/62 (9.68%)	13/103 (12.62%)	5/37 (13.51%)	4/24 (16.67%)	14/129 (10.85%)	14/97 (14.43%)
	OR (95% CI)		Ref.	1.10 (0.34-3.59)	0.70 (0.17-2.98)	1.37 (0.29-6.60)	Ref.	1.07 (0.43-2.68)
	p-value			0.9	0.6	0.7		0.9
**crt I356T**	n/N (%)	770	17/176 (9.66%)	38/299 (12.71%)	25/165 (15.15%)	24/130 (18.46%)	55/409 (13.45%)	49/361 (13.57%)
	OR (95% CI)		Ref.	1.39 (0.68-2.83)	1.17 (0.54-2.51)	**2.36 (1.04-5.34)**	Ref.	1.12 (0.67-1.86)
	p-value			0.4	0.7	**0.04**		0.7
**dhps S436A**	n/N (%)	804	64/180 (35.56%)	101/311 (32.48%)	65/173 (37.57%)	48/140 (34.29%)	135/429 (31.47%)	143/375 (38.13%)
	OR (95% CI)		Ref.	0.98 (0.65-1.47)	1.18 (0.74-1.87)	0.91 (0.56-1.48)	Ref.	1.35 (1.00-1.83)
	p-value			>0.9	0.5	0.7		0.053
**dhps K540E**	n/N (%)	761	69/172 (40.12%)	128/296 (43.24%)	65/166 (39.16%)	69/127 (54.33%)	166/406 (40.89%)	165/355 (46.48%)
	OR (95% CI)		Ref.	1.17 (0.79-1.74)	1.00 (0.64-1.58)	**1.85 (1.14-2.99)**	Ref.	1.31 (0.97-1.77)
	p-value			0.4	>0.9	**0.013**		0.074
**dhps A581G**	n/N (%)	808	6/179 (3.35%)	9/315 (2.86%)	8/175 (4.57%)	8/139 (5.76%)	15/430 (3.49%)	16/378 (4.23%)
	OR (95% CI)		Ref.	0.86 (0.29-2.57)	1.4 (0.45-4.33)	1.82 (0.59-5.66)	Ref.	1.24 (0.58-2.65)
	p-value			0.8	0.6	0.3		0.6
**mdr1 Y184F**	n/N (%)	788	91/178 (51.12%)	145/304 (47.7%)	95/170 (55.88%)	77/136 (56.62%)	228/423 (53.9%)	180/365 (49.32%)
	OR (95% CI)		Ref.	0.87 (0.60-1.27)	1.2 (0.78-1.84)	1.2 (0.76-1.89)	Ref.	0.84 (0.63-1.12)
	p-value			0.5	0.4	0.4		0.2
**mdr2 I492V**	n/N (%)	739	115/170 (67.65%)	168/292 (57.53%)	90/157 (57.32%)	85/120 (70.83%)	251/400 (62.75%)	207/339 (61.06%)
	OR (95% CI)		Ref.	**0.65 (0.43-0.96)**	0.64 (0.41-1.01)	1.15 (0.69-1.92)	Ref.	0.94 (0.70-1.28)
	p-value			**0.031**	0.056	0.6		0.7
**any k13 non-synonymous mutation**	n/N (%)	739	4/180 (2.22%)	8/315 (2.54%)	6/175 (3.43%)	4/140 (2.86%)	12/431 (2.78%)	10/379 (2.64%)
	OR (95% CI)		Ref.	1.11 (0.33-3.82)	1.55 (0.42-5.71)	1.31 (0.31-5.44)	Ref.	0.96 (0.40-2.27)
	p-value			0.9	0.5	0.7		>0.9

Table S11: Mixed-effects logistic regression of genotype outcomes with variables occupation and gender, and random effects for health facilities

			Occupation					Gender	
		**N**	Minor	Student	Agricultural	Other	Unemployed	Female	Male
**crt 72-76 CVIET**	n/N (%)	815	45/277 (16.25%)	59/332 (17.77%)	32/85 (37.65%)	13/84 (15.48%)	5/37 (13.51%)	78/430 (18.14%)	76/385 (19.74%)
	OR (95% CI)		Ref.	0.84 (0.50-1.43)	**2.65 (1.26-5.58)**	1.73 (0.73-4.11)	2.61 (0.75-9.08)	Ref.	1.44 (0.91--2.26)
	p-value			0.5	**0.01**	0.2	0.13		0.12
**crt A220S**	n/N (%)	225	10/96 (10.42%)	13/96 (13.54%)	2/15 (13.33%)	0/12 (0%)	2/6 (33.33%)	13/128 (10.16%)	14/97 (14.43%)
	OR (95% CI)		NA	NA	NA	NA	NA	NA	NA
	p-value		NA	NA	NA	NA	NA	NA	NA
**crt I356T**	n/N (%)	765	30/267 (11.24%)	42/309 (13.59%)	20/76 (26.32%)	7/79 (8.86%)	3/34 (8.82%)	53/405 (13.09%)	49/360 (13.61%)
	OR (95% CI)		Ref.	0.96 (0.53-1.74)	**2.45 (1.05-5.72)**	1.12 (0.39-3.23)	2.66 (0.54-13.0)	Ref	1.26 (0.75-2.12)
	p-value			0.9	**0.038**	0.8	0.2		0.4
**dhps S436A**	n/N (%)	799	96/273 (35.16%)	105/323 (32.51%)	37/82 (45.12%)	25/84 (29.76%)	13/37 (35.14%)	133/425 (31.29%)	143/374 (38.24%)
	OR (95% CI)		Ref.	1.00 (0.69-1.43)	1.29 (0.75-2.22)	0.84 (0.48-1.47)	1.08 (0.51-2.30)	Ref.	**1.43 (1.05-1.94)**
	p-value			>0.9	0.4	0.5	0.8		**0.025**
**dhps K540E**	n/N (%)	756	109/263 (41.44%)	131/310 (42.26%)	39/73 (53.42%)	38/76 (50%)	12/34 (35.29%)	165/402 (41.04%)	164/354 (46.33%)
	OR (95% CI)		Ref.	1.07 (0.75-1.52)	**1.95 (1.11-3.45)**	1.33 (0.78-2.28)	0.65 (0.30-1.42)	Ref.	0.65 (0.30-1.42)
	p-value			0.7	**0.021**	0.3	0.3		0.091
**dhps A581G**	n/N (%)	803	9/273 (3.3%)	13/327 (3.98%)	6/82 (7.32%)	2/84 (2.38%)	1/37 (2.7%)	15/426 (3.52%)	16/377 (4.24%)
	OR (95% CI)		Ref.	0.95 (0.38-2.38)	3.05 (0.93-9.97)	0.78 (0.16-3.86)	1.14 (0.13-9.94)	Ref.	1.32 (0.61-2.88)
	p-value			>0.9	0.065	0.8	>0.9		0.5
**mdr1 Y184F**	n/N (%)	783	134/270 (49.63%)	156/317 (49.21%)	46/79 (58.23%)	48/80 (60%)	19/37 (51.35%)	224/419 (53.46%)	179/364 (49.18%)
	OR (95% CI)		Ref.	0.99 (0.71-1.39)	1.34 (0.79-2.26)	1.53 (0.92-2.54)	1.05 (0.53-2.10)	Ref.	0.86 (0.64-1.14)
	p-value			>0.9	0.3	0.1	0.9		0.3
**mdr2 I492V**	n/N (%)	734	168/260 (64.62%)	173/301 (57.48%)	48/72 (66.67%)	47/68 (69.12%)	18/33 (54.55%)	248/396 (62.63%)	206/338 (60.95%)
	OR (95% CI)		Ref.	0.74 (0.53-1.05)	1.1 (0.62-1.93)	1.24 (0.70-2.22)	0.66 (0.31-1.38)	Ref.	0.94 (0.69-1.28)
	p-value			0.091	0.8	0.5	0.3		0.7
**any k13 non-synonymous mutation**	n/N (%)	734	6/274 (2.19%)	9/327 (2.75%)	5/83 (6.02%)	2/84 (2.38%)	0/37 (0%)	12/427 (2.81%)	10/378 (2.65%)
	OR (95% CI)		NA	NA	NA	NA	NA	NA	NA
	p-value		NA	NA	NA	NA	NA	NA	NA

Table S12: Proportion of samples carrying a *dhps* haplotype. Source data for Figure 3C.

Number of samples carrying the haplotype (% of total)

dhps 431/436/436/437/540/581/613 haplotype		Country	Benguela	Bie	Cuando Cubango	Moxico	Namibe	Uige	Zaire
	N	791	121	131	111	113	65	117	133
	Classification								
ISAKAA	wild type	12 (1.52%)	1 (0.83%)	0	1 (0.9%)	4 (3.54%)	0	4 (3.42%)	2 (1.5%)
IAAKAA	<2 mutations	2 (0.25%)	0	0	0	1 (0.88%)	0	1 (0.85%)	0
IAGKAA	<2 mutations	65 (8.22%)	7 (5.79%)	10 (7.63%)	11 (9.91%)	10 (8.85%)	2 (3.08%)	9 (7.69%)	16 (12.03%)
ISGKAA	<2 mutations	259 (32.74%)	57 (47.11%)	22 (16.79%)	20 (18.02%)	36 (31.86%)	30 (46.15%)	45 (38.46%)	49 (36.84%)
IAAKAA+ISGKAA	<2 mutations	1 (0.13%)	0	0	0	1 (0.88%)	0	0	0
IAGKAA+ISAKAA	<2 mutations	2 (0.25%)	0	0	0	0	0	1 (0.85%)	1 (0.75%)
IAGKAA+ISGKAA	<2 mutations	69 (8.72%)	5 (4.13%)	15 (11.45%)	12 (10.81%)	5 (4.42%)	4 (6.15%)	17 (14.53%)	11 (8.27%)
ISAKAA+ISGKAA	<2 mutations	9 (1.14%)	0	0	0	4 (3.54%)	1 (1.54%)	3 (2.56%)	1 (0.75%)
ISGKAA+ISGKAS	<2 mutations	1 (0.13%)	0	0	0	0	1 (1.54%)	0	0
IAAKAA+IAGKAA+ISGKAA	<2 mutations	1 (0.13%)	0	0	0	0	0	1 (0.85%)	0
IAGKAA+ISAKAA+ISGKAA	<2 mutations	3 (0.38%)	0	1 (0.76%)	1 (0.9%)	0	0	1 (0.85%)	0
IAGEAA	double	2 (0.25%)	0	1 (0.76%)	1 (0.9%)	0	0	0	0
ISGEAA	double	98 (12.39%)	17 (14.05%)	15 (11.45%)	19 (17.12%)	22 (19.47%)	12 (18.46%)	3 (2.56%)	10 (7.52%)
IAGEAA+IAGKAA	double	6 (0.76%)	0	2 (1.53%)	3 (2.7%)	0	0	1 (0.85%)	0
IAGEAA+ISGEAA	double	5 (0.63%)	0	0	3 (2.7%)	1 (0.88%)	1 (1.54%)	0	0
ISGEAA+ISGKAA	double	111 (14.03%)	22 (18.18%)	25 (19.08%)	23 (20.72%)	12 (10.62%)	9 (13.85%)	6 (5.13%)	14 (10.53%)
ISGEAA+VAGEAA	double	1 (0.13%)	0	0	1 (0.9%)	0	0	0	0
ISGEGA	triple	5 (0.63%)	0	0	0	0	0	1 (0.85%)	4 (3.01%)
ISGEGA+ISGKAA	triple	3 (0.38%)	0	0	0	0	0	0	3 (2.26%)
ISGEAA+ISGEGA+ISGKAA	triple	3 (0.38%)	0	0	0	1 (0.88%)	0	0	2 (1.5%)
Undetermined[a]	Undet.	133 (16.81%)	12 (9.92%)	40 (30.53%)	16 (14.41%)	16 (14.16%)	5 (7.69%)	24 (20.51%)	20 (15.04%)

[a]Mixed genotype in more than 2 targets.

Table S13: Proportion of samples carrying a *dhfr* haplotype. Source data for Figure 3C.

Number of samples carrying the haplotype (% of total)

		**Country**	**Benguela**	**Bie**	**Cuando Cubango**	**Moxico**	**Namibe**	**Uige**	**Zaire**
***dhfr* 16/51/59/108/164 haplotype**	**N**	800	124	132	113	115	64	119	133
	**Class.**								
ANCSI	wild type	1 (0.12%)	0	0	0	0	0	0	1 (0.75%)
AICNI	<3 mutations	93 (11.62%)	27 (21.77%)	15 (11.36%)	10 (8.85%)	8 (6.96%)	16 (25%)	6 (5.04%)	11 (8.27%)
ANCNI	<3 mutations	1 (0.12%)	0	0	0	1 (0.87%)	0	0	0
ANRNI	<3 mutations	3 (0.38%)	0	1 (0.76%)	0	2 (1.74%)	0	0	0
AICNI+ANRNI	<3 mutations	3 (0.38%)	2 (1.61%)	0	0	1 (0.87%)	0	0	0
AIRNI	triple	468 (58.5%)	66 (53.23%)	49 (37.12%)	59 (52.21%)	76 (66.09%)	34 (53.12%)	75 (63.03%)	109 (81.95%)
AICNI+AIRNI	triple	195 (24.38%)	26 (20.97%)	55 (41.67%)	39 (34.51%)	19 (16.52%)	12 (18.75%)	33 (27.73%)	11 (8.27%)
AIRNI+ANCNI	triple	1 (0.12%)	0	0	0	1 (0.87%)	0	0	0
AIRNI+ANRNI	triple	18 (2.25%)	2 (1.61%)	5 (3.79%)	3 (2.65%)	5 (4.35%)	2 (3.12%)	0	1 (0.75%)
AICNI+AIRNI+ ANRNI	triple	17 (2.12%)	1 (0.81%)	7 (5.3%)	2 (1.77%)	2 (1.74%)	0	5 (4.2%)	0

Table S14: Proportion of samples carrying a *dhps-dhfr* haplotype. Source data for Figure 3C.

Number of samples carrying the haplotype (% of total)

dhps-dhfr haplotype	N / Class.	Country	Benguela	Bie	Cuando Cubango	Moxico	Namibe	Uige	Zaire
	N	790	121	131	111	113	64	117	133
	Class.								
IAAKAA-AIRNI	<5 mutations	2 (0.25%)	0	0	0	1 (0.88%)	0	1 (0.85%)	0
IAGKAA-AICNI	<5 mutations	14 (1.77%)	2 (1.65%)	2 (1.53%)	4 (3.6%)	1 (0.88%)	1 (1.56%)	0	4 (3.01%)
IAGKAA-AIRNI	<5 mutations	47 (5.95%)	4 (3.31%)	8 (6.11%)	7 (6.31%)	7 (6.19%)	0	9 (7.69%)	12 (9.02%)
IAGKAA-ANRNI	<5 mutations	1 (0.13%)	0	0	0	1 (0.88%)	0	0	0
ISAKAA-AICNI	<5 mutations	4 (0.51%)	0	0	0	2 (1.77%)	0	1 (0.85%)	1 (0.75%)
ISAKAA-AIRNI	<5 mutations	7 (0.89%)	1 (0.83%)	0	1 (0.9%)	1 (0.88%)	0	3 (2.56%)	1 (0.75%)
ISAKAA-ANCNI	<5 mutations	1 (0.13%)	0	0	0	1 (0.88%)	0	0	0
ISGEAA-AICNI	<5 mutations	12 (1.52%)	4 (3.31%)	5 (3.82%)	0	3 (2.65%)	0	0	0
ISGKAA-AICNI	<5 mutations	51 (6.46%)	16 (13.22%)	6 (4.58%)	4 (3.6%)	2 (1.77%)	14 (21.88%)	4 (3.42%)	5 (3.76%)
ISGKAA-AIRNI	<5 mutations	169 (21.39%)	31 (25.62%)	10 (7.63%)	9 (8.11%)	29 (25.66%)	12 (18.75%)	36 (30.77%)	42 (31.58%)
ISGKAA-ANCSI	<5 mutations	1 (0.13%)	0	0	0	0	0	0	1 (0.75%)
ISGKAA-ANRNI	<5 mutations	2 (0.25%)	0	1 (0.76%)	0	1 (0.88%)	0	0	0
IAGKAA-AICNI+ IAGKAA-AIRNI	<5 mutations	3 (0.38%)	1 (0.83%)	0	0	1 (0.88%)	1 (1.56%)	0	0
IAGKAA-AICNI+ ISGKAA-AICNI	<5 mutations	3 (0.38%)	0	0	2 (1.8%)	0	0	0	1 (0.75%)
IAGKAA-AIRNI+ ISAKAA-AIRNI	<5 mutations	1 (0.13%)	0	0	0	0	0	1 (0.85%)	0
IAGKAA-AIRNI+ ISGKAA-AIRNI	<5 mutations	25 (3.16%)	1 (0.83%)	2 (1.53%)	5 (4.5%)	3 (2.65%)	1 (1.56%)	4 (3.42%)	9 (6.77%)
ISAKAA-AIRNI+ ISGKAA-AIRNI	<5 mutations	2 (0.25%)	0	0	0	2 (1.77%)	0	0	0
ISGEAA-AICNI+ ISGKAA-AICNI	<5 mutations	3 (0.38%)	2 (1.65%)	1 (0.76%)	0	0	0	0	0
ISGKAA-AICNI+ ISGKAA-AIRNI	<5 mutations	29 (3.67%)	6 (4.96%)	5 (3.82%)	7 (6.31%)	2 (1.77%)	4 (6.25%)	4 (3.42%)	1 (0.75%)
ISGKAA-AICNI+ ISGKAA-ANRNI	<5 mutations	3 (0.38%)	2 (1.65%)	0	0	1 (0.88%)	0	0	0
ISGKAA-AICNI+ ISGKAS-AICNI	<5 mutations	1 (0.13%)	0	0	0	0	1 (1.56%)	0	0
ISGKAA-AIRNI+ ISGKAA-ANRNI	<5 mutations	3 (0.38%)	2 (1.65%)	0	0	1 (0.88%)	0	0	0
ISGKAA-AICNI+ ISGKAA-AIRNI+ ISGKAA-ANRNI	<5 mutations	1 (0.13%)	0	0	0	0	0	1 (0.85%)	0
IAGEAA-AIRNI	quintuple	2 (0.25%)	0	1 (0.76%)	1 (0.9%)	0	0	0	0
ISGEAA-AIRNI	quintuple	77 (9.75%)	13 (10.74%)	6 (4.58%)	17 (15.32%)	18 (15.93%)	10 (15.62%)	3 (2.56%)	10 (7.52%)
IAGEAA-AIRNI+ IAGKAA-AIRNI	quintuple	4 (0.51%)	0	2 (1.53%)	1 (0.9%)	0	0	1 (0.85%)	0
IAGEAA-AIRNI+ ISGEAA-AIRNI	quintuple	2 (0.25%)	0	0	1 (0.9%)	1 (0.88%)	0	0	0
ISGEAA-AICNI+ ISGEAA-AIRNI	quintuple	5 (0.63%)	0	3 (2.29%)	1 (0.9%)	0	1 (1.56%)	0	0

ISGEAA-AIRNI+ ISGEAA-ANRNI	quintuple	2 (0.25%)	0	1 (0.76%)	0	0	1 (1.56%)	0	0
ISGEAA-AIRNI+ ISGKAA-AIRNI	quintuple	56 (7.09%)	9 (7.44%)	10 (7.63%)	10 (9.01%)	4 (3.54%)	6 (9.38%)	4 (3.42%)	13 (9.77%)
ISGEAA-AIRNI+ VAGEAA-AIRNI	quintuple	1 (0.13%)	0	0	1 (0.9%)	0	0	0	0
ISGEAA-AICNI+ ISGEAA-AIRNI+ ISGEAA-ANRNI	quintuple	2 (0.25%)	0	0	1 (0.9%)	1 (0.88%)	0	0	0
ISGEGA-AIRNI	sextuple	5 (0.63%)	0	0	0	0	0	1 (0.85%)	4 (3.01%)
ISGEGA-AIRNI+ ISGKAA-AIRNI	sextuple	3 (0.38%)	0	0	0	0	0	0	3 (2.26%)
ISGEAA-AIRNI+ ISGEGA-AIRNI+ ISGKAA-AIRNI	sextuple	2 (0.25%)	0	0	0	0	0	0	2 (1.5%)
Undetermined[a]	Undet.	244 (30.89%)	27 (22.31%)	68 (51.91%)	39 (35.14%)	30 (26.55%)	12 (18.75%)	44 (37.61%)	24 (18.05%)

[a]Mixed genotype in more than 2 targets.

Table S15: Proportion of samples carrying an *mdr1* haplotype.

Number of samples carrying the haplotype (% of total)

	**Country**	**Benguela**	**Bie**	**Cuando Cubango**	**Moxico**	**Namibe**	**Uige**	**Zaire**
***mdr1* 86/184/1246 haplotype**	N=649	105	107	73	91	51	94	128
NFD	128 (19.72%)	25 (23.81%)	16 (14.95%)	7 (9.59%)	19 (20.88%)	18 (35.29%)	18 (19.15%)	25 (19.53%)
NFD+NYD	197 (30.35%)	26 (24.76%)	41 (38.32%)	20 (27.4%)	33 (36.26%)	13 (25.49%)	30 (31.91%)	34 (26.56%)
NFD+YFD	1 (0.15%)	0	0	0	0	0	0	1 (0.78%)
NYD	308 (47.46 %)	53 (50.48%)	48 (44.86%)	44 (60.27%)	37 (40.66%)	19 (37.25%)	44 (46.81%)	63 (49.22%)
NYD+YYD	3 (0.46%)	0	0	1 (1.37%)	0	0	0	2 (1.56%)
YFD	1 (0.15%)	0	0	0	0	0	0	1 (0.78%)
YFD+YYD	1 (0.15%)	0	0	0	0	0	1 (1.06%)	0
YYD	3 (0.46%)	0	1 (0.93%)	0	0	1 (1.96%)	0	1 (0.78%)
Undetermined[a]	7 (3.1%)	1 (0.95%)	1 (0.93%)	1 (1.37%)	2 (2.2%)	0	1 (1.06%)	1 (0.78%)

[a]Mixed genotype in more than 2 targets.
